# Rational Design and Synthesis of New, High Efficiency, Multipotent Schiff Base-1,2,4-triazole Antioxidants Bearing Butylated Hydroxytoluene Moieties

**DOI:** 10.3390/molecules21070847

**Published:** 2016-06-28

**Authors:** Wageeh A Yehye, Noorsaadah Abdul Rahman, Omar Saad, Azhar Ariffin, Sharifah Bee Abd Hamid, Abeer A. Alhadi, Farkaad A. Kadir, Marzieh Yaeghoobi, Abdulsalam A. Matlob

**Affiliations:** 1 Nanotechnology & Catalysis Research Centre (NANOCAT), University of Malaya, Block 3A, Institute of Postgraduate Studies Building, Kuala Lumpur 50603, Malaysia; sharifahbee@um.edu.my; 2 Department of Chemistry, Faculty of Science, University of Malaya, Kuala Lumpur 50603, Malaysia; noorsaadah@um.edu.my (N.A.R.); azhar70@um.edu.my (A.A.); aaalhamadani@yahoo.com (A.A.A.); m.yaeghoobi@gmail.com (M.Y.); 3 Drug Design and Development Research Group, Department of Chemistry, Faculty of Science, University of Malaya, Kuala Lumpur 50603, Malaysia; 4 Department of Pharmacy, Faculty of Medicine, University of Malaya, Kuala Lumpur 50603, Malaysia; omar79@siswa.um.edu.my; 5 Division of Human Biology, School of Medicine, International Medical University, Kuala Lumpur 57000, Malaysia; FarqadAbdulhadi@imu.edu.my; 6 Department of Environmental Technology, College of Environment, Mosul University, Mosul 41001, Iraq; matlob58@yahoo.com

**Keywords:** butylated hydroxytoluene, PASS, 1,2,4-triazole, Schiff base, DPPH assay

## Abstract

A new series of multipotent antioxidants (MPAOs), namely Schiff base-1,2,4-triazoles attached to the oxygen-derived free radical scavenging moiety butylated hydroxytoluene (BHT) were designed and subsequently synthesized. The structure-activity relationship (SAR) of the designed antioxidants was established alongside the prediction of activity spectra for substances (PASS). The antioxidant activities of the synthesized compounds **4**–**10** were tested by the DPPH bioassay. The synthesized compounds **4**–**10** inhibited stable DPPH free radicals at a level that is 10^−4^ M more than the well-known standard antioxidant BHT. Compounds **8**–**10** with *para*-substituents were less active than compounds **4** and **5** with trimethoxy substituents compared to those with a second BHT moiety (compounds **6** and **7**). With an IC_50_ of 46.13 ± 0.31 µM, compound **6** exhibited the most promising in vitro inhibition at 89%. Therefore, novel MPAOs containing active triazole rings, thioethers, Schiff bases, and BHT moieties are suggested as potential antioxidants for inhibiting oxidative stress processes and scavenging free radicals, hence, this combination of functions is anticipated to play a vital role in repairing cellular damage, preventing various human diseases and in medical therapeutic applications.

## 1. Introduction

Regulation of reducing and oxidizing status is critical for the viability of cell organ functions [1]. Reactive oxygen species (ROS) have a strong disruptive effect on industrial products, such as oils, plastics and rubbers. They exist in different forms, such as hydroxyls (HO•), superoxide anion radicals (O_2_•-), peroxyls (ROO•), alkoxyls (RO•), and nitric oxides (NO•) [2]. They can potentially cause oxidation in foodstuffs [3], pharmaceuticals [4,5,6], and petroleum and rubber products [7] via several degradation reactions, both upon heating and during long-term storage. The presence of ROS in biological systems was confirmed around 50 years ago [8].

Inflammation and increased oxidative stress contribute actively to increased pathogenesis of many human diseases due to undesirable processes, such as aging [9], inflammation [10,11] and many others [12,13,14,15,16].

Pharmacological strategies should aim to supplement antioxidant defense systems while antagonizing redox-sensitive signal transduction, which will subsequently lead to improved clinical management of the inflammatory process [17]. Antioxidants also represent a wide class of additives used for food [18], lubricants [19], polymers [20] and paints [21], etc.

Phenolic antioxidants form a significant class of compounds both commercial and biological importance, which serve to inhibit oxidation to counteract the autoxidation reaction of hydrocarbons, unsaturated fatty acids or esters, and other substances [22]. Synthetic antioxidants, particularly phenols that are used as food, polymer, pharmaceutical, and oil additives, include BHT, butylated hydroxyanisole (BHA) and *tert*-butylhydroquinone [23]. BHT [24] has been approved by FDA since 1954 [23] for use in food packaging. Currently, BHT is the most commonly used antioxidant in foods [25], rubber [7] and pharmaceutical products. Due to its industrial applications, researchers have designed and synthesized a series of BHT derivatives to accelerate the antioxidant discovery process, aiming to expand its applications with a wide range of antioxidant activities to markedly enhance the radical scavenging ability and other physical properties. Accordingly, some rational-design strategies for antioxidants based on BHT structure have been designed and the corresponding target compounds synthesized [26,27,28].

1,2,4-Triazoles are a class of five membered heterocycles, extensively researched due to their medicinal properties [29,30]. 1,2,4-Triazole-5-thiones are known for their anti-inflammatory [31], COX-2 inhibitor [32], and antimycotic [33] activities. A series of Schiff bases of 3-substituted-1,2,4-triazo-5-thione have been synthesized and evaluated for their antioxidant activity using the hydrogen peroxide scavenging method [34].

In this study, SAR and rational design approaches were utilized to integrate multiple radical-scavenging activities in designing hybrid compounds with significantly enhanced radical-scavenging capacity. These approaches were verified with PASS training subsets. PASS-predicted antioxidant activities for Schiff base-1,2,4-triazoles bearing BHT moieties were experimentally verified using DPPH assays.

## 2. Results and Discussion

### 2.1. SAR and Designing of MPAO

**Figure 1:**
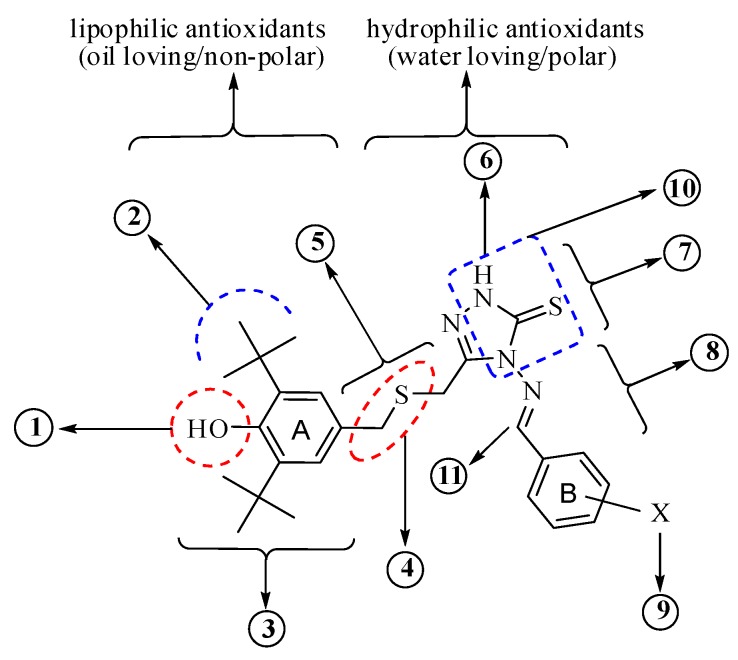
Rational design of Schiff base-1,2,4-triazoles bearing BHT moieties.

The design approach for free radical inhibitors involves gathering the useful features of different antioxidant fragments into a single structure. This approach was implemented to increase the antioxidant activities and other physical properties of the well-known antioxidant (BHT) and identify novel MPAOs bearing particular antioxidant fragments that are very important in the introduction of promising MPAO candidates possessing different physical and chemical properties, as illustrated in Figure 1. These features include the following:

### 2.2. PASS—Prediction

PASS tools are constructed to predict over 4000 types of biological activity, including drug and non-drug actions. PASS predictions can be used to recognize the most probable targets at the molecular level [35], with 90% accuracy [36,37,38,39]. It has been reported that PASS predictions can be successfully applied and confirmed by pharmacological research experiments [35,40,41,42]. PASS results are presented as a list with an appropriate Pa (compound probability to be active) and Pi (compound probability to be inactive). Compounds with Pa > Pi are assumed to be active. For example, if Pa > 0.7, the possibility of discovering the activity in the experiment is high, but in some cases, the compound’s structure is analogous to the PASS training set. If 0.5 < Pa < 0.7, the possibility of discovering the activity in the experiment is less, however, the compound’s structure is dissimilar to the PASS training set. If Pa < 0.5, the possibility of finding the activity in the experiment is even less, but if it is verified, then there is more than 50% possibility that this structure has not been reported in the context of this particular biological activity, and this might be a valuable lead compound.

The PASS approach has significant limitations: (i) the activity spectrum cannot be predicted for compounds that have no identifier in the PASS training set; and (ii) PASS predictions can only clarify the intrinsic activity of a compound via experiments. In this work PASS was used to predict the biological activities of Schiff base-1,2,4-triazoles bearing BHT moieties to promote the search for high efficiency new MPAOs. The potential antioxidant activities of the designed compounds were predicted based on SAR analysis of the PASS training set from many sources, including publications, patents, databases, and private communications.

Therefore, PASS predicted different antioxidant activities for the synthesized compounds, such as lipid peroxidase inhibition, antioxidant, and free radical scavenging, and BHT, as given in Table 1. Antioxidant activities generated by PASS were validated by DPPH bioassays.

### 2.3. Synthesis

**Scheme 1:**
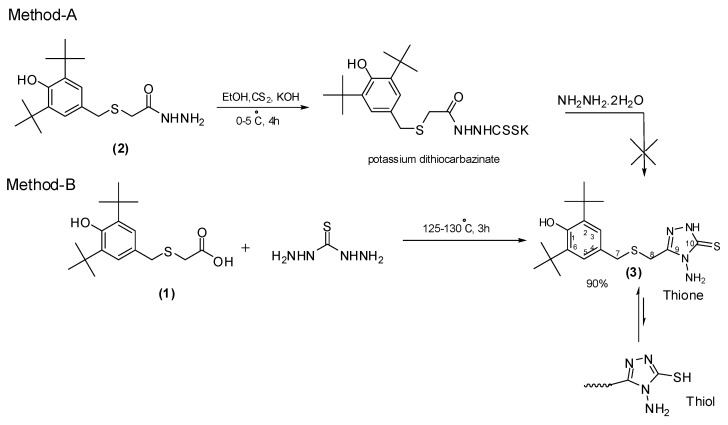
Synthetic scheme for the formation of 4-amino-3-((3,5-di-*tert*-butyl-4-hydroxybenzylthio)methyl)-1*H*-1,2,4-triazole-5(4*H*)-thione (**3**).

The synthesis of the designed compounds **3**–**10** is outlined in Scheme 1. 2-(3,5-di-*tert*-Butyl-4-hydroxybenzylthio)acetic acid (**1**) and corresponding acid hydrazide **2** were synthesized as previously described [26]. The 4-amino-1,2,4-triazole system is a heterocyclic organic compound that consists of a 1,2,4-triazole substituted with an amino group. A synthetic approach to compound **3** is outlined in Scheme 1. The most common synthetic approach reported for the preparation of 4-amino-1,2,4-triazole rings is via the formation of potassium dithiocarbazinate, which is then reacted with hydrazine hydrate in an aqueous solution (Scheme 1, Method-A) [43,44]. However, in our hands, the desired product was not obtained when this method was employed. It is believed that the reaction product underwent hydrolysis, resulting in a mixture of products being obtained according to TLC.

We then attempted a different method (Scheme 1, Method-B) by fusing compound **1** with thiocarbohydrazide at different mole ratios, as well as different temperature ranges. The results are summarized in Table 2.

Carrying out the reaction with a mole ratio of 1:1 of compound **1** to thiocarbohydrazide (entry 1) resulted in a very low yield (24%). Altering the mole ratio of **1** to thiocarbohydrazide to 1:1.25 (entry 2) resulted in a much higher yield (70%). Increasing the reaction temperature to 155–160 °C resulted in a decrease in the yield to only 45% (entry 3). When a 1:1.5 mole ratio was used (entry 4), a satisfactory yield of 90% was obtained. However, if the 1:2 mole ratio was used, the yield did not increase apparently (entry 5), while increasing the reaction temperature in entry 4 decreased the yield (entry 6).

Formation of compounds **3**–**10** was confirmed by recording their IR, NMR, high-resolution mass spectra. The IR spectra of compounds **3**–**10** showed absorption bands at 3639, (3192, 3269), 1629, and 1231 cm*^−^*^1^ due to the O–H, N–H, C=N and C=S groups, respectively. In the ^1^H-NMR spectrum of **3**, we find a sharp singlet peak at 1.40 ppm, due to the eighteen equivalent methyl protons of 2-C(CH_3_)_3_. Thioether group H-7 and H-8 exhibited two singlets (A_2_ system) at 3.63 ppm and 3.64 ppm, respectively. The singlet at 4.74 ppm, with the integration of two protons, corresponds to the –NH_2_ group. The singlet peak at 5.18 ppm is attributed to the O–H of the hindered phenol. A singlet peak at 7.11 ppm with the integration of two protons was assigned to the two symmetrical aromatic ring protons, H-3 and H-5. The NH-proton of the triazole ring resonated as a broad band at 11.38 ppm, which agrees with the presence of the thione form (see the ^1^H-NMR spectrum of **3** in the Supplementary Materials). The ^13^C-NMR spectra of **3** confirmed the presence of two sp^2^ methines (C-3 and C-5), eight quaternary carbons. C-2 and C-6 are symmetrical, and attached to di-*tert*-butyl groups. The quaternary carbons C-9 and C-10 appeared in the region at 150.42 and 167.06 ppm, respectively (see the ^13^C-NMR spectrum of **3** in the Supplementary Materials).

**Scheme 2:**
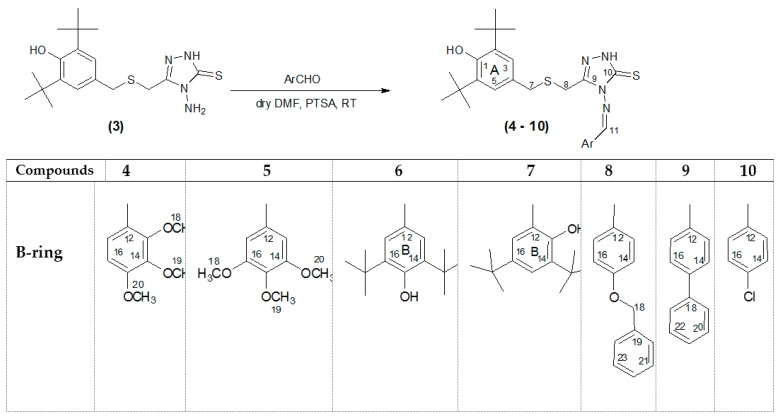
Synthetic scheme for the formation of 4-(substituted benzylideneamino)-3-((3,5-di-*tert*-butyl-4-hydroxybenzyl thio)methyl)-1*H*-1,2,4-triazole-5(4*H*)-thiones **4**–**10**.

The condensation reaction of **3**, with substituted aromatic aldehydes in the presence of PTSA in DMF at RT, resulted in a good yields of products, ranging from 66%–85% (Scheme 2).

**Figure 2:**
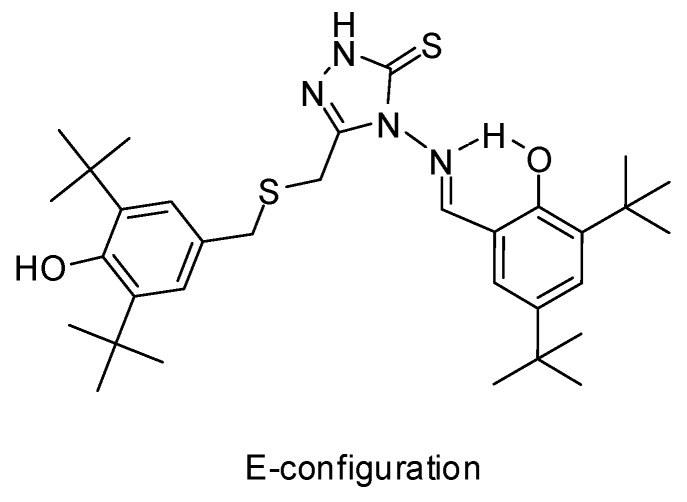
Proposed intra-molecular hydrogen bonding of **7**.

The ^1^H-NMR spectra of **4**–**10** showed a singlet peak between 1.41–1.42 ppm, due to the eighteen equivalent protons of 2-C(CH_3_)_3_, while the thioether group H-7 and H-8 exhibits two very close singlets in the region 3.70–3.72 and 3.65–3.75 ppm, respectively. Two protons appeared as a singlet between 7.09–7.12 ppm, which is assigned to the two symmetrical aromatic ring protons H-3 and H-5. The hydroxyl group link to C-1 of all the synthesized Schiff base-1,2,4-triazoles show a singlet peak in the region 5.15–5.17 ppm, due to the free O–H, which is flanked by two *tert*-butyl groups, while only **6** and **7** showed other phenolic hydroxyl functional groups (see the ^1^H-NMR spectra of **4**–**10** in the Supplementary Materials). In **6**, a singlet at 5.70 ppm was assigned to the phenolic OH linked to C-15, which was also flanked by two *tert-*butyl groups. In contrast to **6**, the phenolic OH of **7**, which is linked to C-13, is significantly deshielded to 10.61 ppm, due to its lesser steric effect than that of an O–H between the two bulky groups, which supports the fact that the azomethine bond has an *E*-configuration, while the hydroxy group bears a hydrogen atom, which is a donor in an intramolecular H bond to the azomethine nitrogen atom [45] (Figure 2).

Schiff base-1,2,4-triazoles **4**–**10** show singlet peaks in the region between 10.60 to 10.33 ppm, due to a NH, indicating the presence of a triazole ring, while the other singlets between 9.87–10.43 ppm were attributed to CH=N, and the disappearance of the NH_2_ protons of **3** further confirmed the presence of a five-membered ring, as well as an imine system present within one molecule. The ^13^C-NMR spectra of **4**–**10** indicated the presence of one sp^2^ methine shifted downfield between 159.07–168.69 ppm. The complete assignments for the ^13^C-NMR spectra of compounds **4**–**10** are illustrated in the Supplementary Materials.

### 2.4. In Vitro DPPH Radical Scavenging Activity

To verify the antioxidant activity of the designed MPAO, an in vitro DPPH antioxidant assay was used. Schiff base-1,2,4-triazoles **4**–**10** were examined for their antioxidant potency using a well-known standard antioxidant, BHT. In its radical form, DPPH• absorbs at 570 nm. DPPH• is scavenged by an antioxidant through hydrogen atom transfer reactions to form a stable DPPH molecule, resulting in a color change from purple to yellow [46,47].

The antioxidant activities of various phenolic compounds are determined by monitoring the concentration of the highly coloured and stable radical DPPH•, which has an unpaired valence electron at one of the nitrogen bridge in the context of spectrometry [46,47]. The ability of DPPH to capture free radicals could be due to the delocalization of the unpaired electron over the DPPH structure [48]. The delocalization of the unpaired electron over the DPPH structure showed its ability to react with different free radicals at three different stages: (i) at the *p*-position on the phenyl ring; (ii) the nitrogen-centered radical and (iii) on the picryl ring [48].

In the DPPH free radical assay, antioxidant efficiency is measured at ambient temperature, thus eliminating the risk of thermal degradation of the molecules being tested [49]. However, the DPPH method also has its limitations, particularly since the nitrogen-centered radical portion of the DPPH structure is accessible only to smaller molecules. Larger molecules may have limited access, due to steric hindrance [50]. Steric accessibility of an antioxidant compound determines the type of reaction mechanisms [51]. Small molecules that are exposed are in better contact with the nitrogen-centered radical site, showing higher antioxidant activity. Since DPPH• is a stable nitrogen radical, unlike the highly reactive and transient peroxyl radicals, which is involved in lipid peroxidation, many phenolic antioxidants that react quickly with peroxyl radicals may react slowly with, or may even be inert to DPPH•, due to the steric inaccessibility effects [51]. Bondet et al. [49] reported that most phenolic antioxidants slowly react with DPPH, reaching steady state in 1–6 h, or longer. It was also reported that the reaction of DPPH• is reversible with some phenols. This would result in false low readings for antioxidant capacity of samples containing phenols [49]. Furthermore, a color interference of DPPH with samples that contain anthocyanins led to the underestimation of the antioxidant activity [52].

**Scheme 3:**
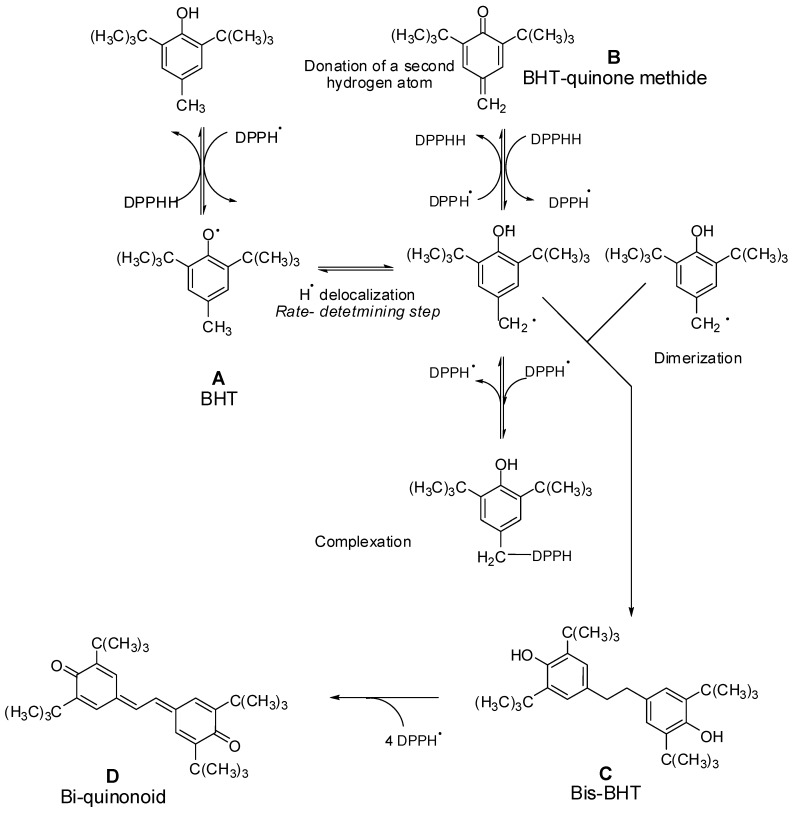
Proposed mechanism for BHT/DPPH• reaction.

Compounds studied in this work were designed based-on antioxidant activity of the BHT moiety. BHT is one of the control groups in the experiment, and it would therefore be useful to understand the plausible mechanisms involving BHT and DPPH•. The reaction mechanism between BHT and DPPH radical is believed to follow three possible pathways, as demonstrated in Scheme 3 [49]. The reaction of BHT with DPPH• form a BHT radical species **A**, which can form compound **B** via the donation of a second hydrogen atom, or dimerizing to form bis-BHT, **C**, which presumably reacted for the second time with DPPH• to produce the bi-quinonoid compound **D** [53].

Most synthesized Schiff base-1,2,4-triazoles that were tested significantly inhibited the DPPH• levels compared to the standard antioxidant (BHT) used in the study (Table 3). This might be due to the presence of more than one antioxidant functional group within the structure of the Schiff base-1,2,4-triazoles. Our synthesized molecules have similar active groups, but different antioxidant activity against DPPH radicals, due to the different substituents attached to the B ring. Therefore, the main source of antioxidant activity of compounds **4**–**10** are strongly attributed to the following antioxidant functional groups presented in our design.

#### 2.4.1. BHT Moiety (Primary Antioxidant)

**Figure 3:**
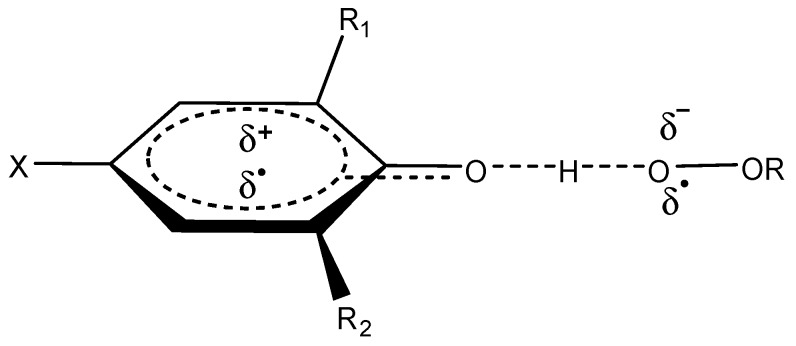
Transition state of hydrogen abstraction by ROS.

It has been reported that substituents, such as *tert*-butyl on the *ortho* and *para* positions increase the antioxidant activity of phenols [54] due to the lowering of the O-H bond dissociation energy (BDE) [55] and the stabilization of the phenoxyl radical by inductive and hyperconjugative effects. This can be rationalized on the basis of the transition state shown in Figure 3, where there is a partial separation of charge between the alkylperoxyl and aromatic ring and partial delocalization of the electron in the π-bond. Placing electron-releasing group that can delocalize unpaired electrons in R_1_, R_2_, and X will decrease the transition state energy [56,57].

**Figure 4:**
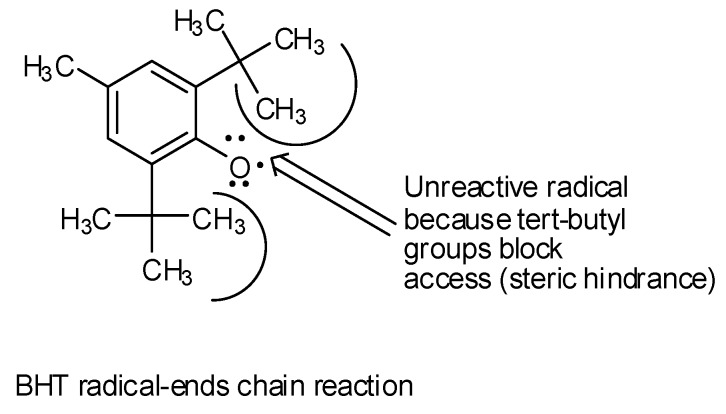
Steric hindrance effects on stabilization of phenoxyl radical [57].

Furthermore, substituents at the o-position would cause the steric hindrance to reduce pro-oxidation reactions [58,59], as shown in Figure 4.

Thus, the reactions of BHT moiety of **A** ring with DPPH• led to hydrogen abstraction from the O–H bond via delocalization of the oxygen electron pair over the aromatic ring, forming stable phenoxyl radical. Therefore, the BHT moiety of the A ring was chosen to be a chain-breaking antioxidant unit in the designs of MPAO Figure 1. This phenomenon could improve the antioxidant activity of our newly designed molecules, causing it to perform at a similar level to the standard antioxidant agent (BHT).

#### 2.4.2. Thioether Function (Secondary Antioxidant)

Thioethers are frequently referred to as hydroperoxide decomposers, and are therefore classified as secondary antioxidants [59]. Thioethers undergo redox reactions with hydroperoxides to form non-radical stable products [56,60]. Sulfur-containing phenols are of particular interest, as they can behave synergistically as chain-breaking and preventive antioxidants by decomposing hydroperoxides to the corresponding alcohols via nucleophilic attack of sulfur on oxygen [61]. Moreover, sulfur is considered to be more effective than oxygen at stabilizing a neighbouring radical center due to better lone-pair interaction with the carbon *p*-orbital [62]. Sulfur containing phenols might be better antioxidants than the phenols containing oxygen substituents at the *para* position to the phenolic group. Therefore, the designed compounds contain thioether antioxidant-bridge as its linkage between the BHT moiety (A ring) and other antioxidants, as well as its functionalities.

#### 2.4.3. Triazole-5-thione Ring

**Figure 5:**
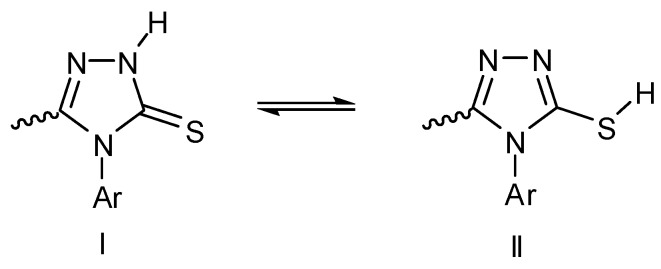
Thione I and thiol II tautomers of 4-amino-1,2,4-triazol-5-thione.

Triazole-5-thione is present as a planar molecule in the thione form (I) over the thiol form (II) (Figure 5). The sulfur atom was the atom with the largest electron density in the molecule [63]. Thus, the chemical behavior of the system towards free radicals would depend on the tautomeric form present in the reaction, assuming that the sulfur atom is involved with the reactive site. It was found that the triazole-5-thione derivatives are relatively more active than the derivatives of thiadiazole, which is due to the presence of thiourea system in triazole ring [57,64,65,66].

Therefore, the presence of triazole-5-thione in our designed compounds could provide a new series of antioxidants for disease prevention and treatment in the field of oxidative stress.

#### 2.4.4. Effect of Ring B Substituents on Antioxidant Activity

The only difference between the designed Schiff base-1,2,4-triazoles **4**–**10** is the nature of the substituents attached to their **B** rings. It has been reported that the electron delocalization across the aromatic imine (**B** ring) and triazole-5-thione attached directly to the N-4 of ring is not an option [66], therefore; the substituents on the phenyl **B** ring and its corresponding imine group, as an aromatic system, are influential upon the radical-scavenging effects, due to their ability to extend the delocalization of free radical and enhance antioxidative abilities. However, according to the nature of their substituents on the **B** ring, the antioxidant activities of the Schiff base-1,2,4-triazoles synthesized in this study can be divided into three classes:

The first class consists of compounds **6** and **7**, which show significant inhibition of DPPH radicals compared to the BHT used in this study as a reference. As shown in Table 3, Schiff base-1,2,4-triazoles **6** and **7** exhibited strong scavenging effect on DPPH, with an IC_50_ value of 46.13 ± 0.31 (inhibition = 89.52%) and IC_50_ value of 52.80 ± 0.58 (inhibition = 79.61% ± 0.23%) μM/mL, respectively, which are thrice better than the BHT with % inhibition of 25.23. At their **B** rings, these compounds contain a phenolic hydroxyl group at the *para* (compound **6**) and *ortho* (compound **7**) positions to the imine group and the electron donating groups (EDGs) at the *ortho*-positions to their hydroxyl groups. The high activity can be explained by the resonance-based stabilizing effects of the EDG and the hydrazone on the phenoxy radical at the **B** ring. The higher activity of compound **6** is due to the enhanced steric hindrance effect provided by the two *ortho*-tertiary butyl groups, which stabilize the phenolic antioxidant radical. In compound **7**, it is believed that an intramolecular hydrogen bond between the phenolic hydrogen with the imine nitrogen disfavors the DPPH scavenging activity of **7**, owing to the enhanced binding of the phenolic hydrogen atom [47], as displayed in Figure 2. It was found that the H-bond stabilizes phenols, therefore, the energy required to extract the hydrogen atom from the O–H is larger than in the non H-bonded phenols [67]. Thus, the OH group of compound **7** exhibited strong hydrogen bonds with the imine group (Figure 2), the O–H, and the N atom of C=N, which could in theory increase the BDE by stabilizing the amine form, making the approach of peroxyl radicals to the O-H group more difficult for steric reasons.

The second class consists of compounds **4** and **5**, showing significant levels of inhibition of DPPH radical compared to the standard antioxidant (BHT), yielding a DPPH• inhibition of 68.13% for **4** and 74.32% for **5**, while BHT resulted in a value of 25.23%. Neither of these compounds contains a phenolic hydroxyl group on the B ring, but they may increase the phenyl ring stabilization, due to formation resonance of stabilized radicals [6].

The third class covers compounds **8**–**10**. All of these compounds contain different substituents at the *para* position to the imine group. The DPPH radical scavenging activity for this class is substantially lower than that of the first and second classes, but better than the standard antioxidant (BHT), due to their resonance-based stabilizing effects [68].

## 3. Materials and Methods

### 3.1. General Information

All materials and solvents were obtained from Sigma–Aldrich (St. Louis, MO, USA). Melting points were determined on a MEL-TEMP II melting point instrument (Barnstead/Thermolyne Corp, North Sioux, SD, USA). IR spectra were recorded on a RX1FT-IR spectrometer (Perkin–Elmer, Waltham, MA, USA). The ^1^H- (400 MHz) and ^13^C-NMR (100 MHz) spectra were recorded on a 400 MHz FT-NMR (JEOL Ltd., Tokyo, Japan) using CDCl_3_ as a solvent and tetramethylsilane as an internal standard. The abbreviations s = singlet, d = doublet, t = triplet, q = quadruplet, m = multiplet and bs = broad signal were used throughout. HR-mass spectra (ESI) were obtained with a Finnigan MAT95XL mass spectrometer at 70 eV (Thermo Fisher Scientific, Waltham, MA, USA). UV-visible spectra were recorded on a model UV-1650PC UV-visible spectrophotometer (Shimadzu Europe, Duisburg, Germany). The Supplementary Data section reports the ^1^H-NMR (Figures S1–S8) and ^13^C-NMR (Figures S9 and S10) spectral data of compounds **3**–**10**.

### 3.2. Synthesis of 4-Amino-3-((3,5-di-tert-butyl-4-hydroxybenzylthio)methyl)-1H-1,2,4-triazole-5(4H)-thione *(**3**)*

#### 3.2.1. Method-A

##### Potassium Dithiocarbazinate Method

Potassium hydroxide (2 mmol, 0.11 g) was dissolved in absolute ethanol (15 mL). *S*-(3,5-di-*tert*-butyl-4-hydroxybenzyl)thioglycolic acid hydrazide (**2**, 1.5 mmol, 0.48 g) was added and the solution was cooled in ice. To this mixture, carbon disulfide (2 mmol, 0.15 g) was added in small portions with constant stirring. The reaction mixture was agitated continuously for a period of 4 h at room temperature. It was then diluted with anhydrous ether. The precipitated potassium dithiocarbazinate was collected by filtration and dried under vacuum. The potassium salt thus obtained was in yellow solid, yield 63% (0.55 g) and was used in the next step without further purification.

A suspension of potassium dithiocarbazinate (0.57 mmol, 0.25 g) in water (5 mL) and hydrazine hydrate (1.71 mmol, 0.08 mL) was stirred for 1 h. The color of the reaction changed to green with the evolution of hydrogen sulfide gas (lead acetate paper and odor). A homogenous reaction mixture was obtained during the reaction process. The reaction mixture was diluted with distilled water (15 mL). On acidification with dilute hydrochloric acid, no triazole **3** was isolated.

#### 3.2.2. Method-B

##### Fusion Reaction Method

*S*-(3,5-di-*tert-*butyl-4-hydroxybenzyl)thioglycolic acid (**1**; 3.10 g, 10 mmol) in a flat bottomed flask was heated in oil bath (100–120 °C) until the acid melted. Thiocarbohydrazide (1.60 g, 15 mmol) was added with stirring. The mixture was maintained at (125–130 °C) for 1 h. Then it was allowed to cool to room temperature and treated with dilute sodium bicarbonate solution in order to remove any unreacted acid. The crude was dissolved in chloroform then filtered to remove unreacted thiocarbohydrazide. The crude product, obtained after concentration under reduced pressure. The product was recrystallised from ethanol to give 3.42 g of white solid (90%). Mp 192–194 °C. IR (KBr), cm^−1^: ν= 3639 (free OH, BHP), 3192, 3269 (NH), 2870–2958 (C–H of *t*-Bu), 1629 (C=N), 1231 (C=S). ^1^H-NMR δ, ppm (Figure S1): 1.40 (s, 18H, 2 × *t-*Bu), 3.63 (s, 2H, H-7), 3.64 (2H, H-8), 4.74 (2H, NH_2_), 5.18 (1H, OH, BHP), 7.11 (s, 2H, H-3, H-5), 11.38 (1H, NH, triazole). ^13^C-NMR δ, ppm (Figure S9): 24.07 (1C, C-8), 30.38 (6C, 2 × –C(CH_3_)_3_, 34.45 (2C, 2 × –C(CH_3_)_3_), 36.26 (1C, C-7), 125.99 (2C, C-3, C-5), 127.19 (1C, C-4), 136.21 (2C, C-2, C-6), 150.42 (1C, C-9), 153.21 (1C, C-1), 167.06 (1C, C-10). HREIMS *m*/*z* 380.1720 [M]^+^ (calcd for C_18_H_28_ON_4_S_2_ 380.1705).

### 3.3. General Procedure for the Synthesis of 4-(Substituted benzylideneamino)-3-(3,5-di-tert-butyl-4-hydroxybenzyl thio) methyl)-1H-1,2,4-triazole-5(4H)-thiones ***4**–**10***

Compound **3** (0.63 mmol, 0.24 g) in DMF (5 mL) was added the appropriate aldehyde (0.63 mmol). A little bit of PTSA was added as a catalyst and the reaction mixture was stirred under nitrogen gas at room temperature for 3 h. The mixture was poured into ice-water (250 mL) with stirring for 3 h. The precipitated solid was filtered off, washed with water to remove the acid catalyst and solvent, dried under vacuum and recrystallized from appropriate solvent.

*3-((3,5-Di-tert-butyl-4-hydroxybenzylthio)methyl)-4-(2,3,4-trimethoxybenzylideneamino)-1H-1,2,4-triazole-5(4H)-thione* (**4**). Prepared from 2,3,4-trimethoxybenzaldehyde (0.63 mmol, 0.123 g), and recrystallized from 5:1 methanol-water to give 0.26 g of white solid (73%). Mp 161–163 °C. IR (KBr), cm^−1^: ν = 3641 (free OH, BHP), 2908–3048 (C–H of CH_3_), 1589 (C=N), 1230 (C=S). ^1^H-NMR δ, ppm (Figure S2): 1.41 (s, 18H, 2 × *t*-Bu), 3.71 (s, 4H, H-7, H-8), 3.88 (3H, H-20, –OCH_3_), 3.922 (3H, H-18, –OCH_3_), 4.00 (3H, H-19, -OCH_3_), 5.16 (1H, OH, BHP), 6.75 (d, 1H, H-17, *J* = 8.75 Hz), 7.10 (s, 2H, H-3, H-5), 7.78 (d, 1H, H-16, *J* = 8.75 Hz), 10.30 (1H, C-11), 11.04 (1H, NH). ^13^C-NMR δ, ppm (Figure S10) : 24.39 (1C, C-8), 30.36 (6C, 2 × –C(CH_3_)_3_, 34.42 (2C, 2 × –C(CH_3_)_3_), 36.53 (1C, C-7), 56.28 (1C, C-18, –OCH_3_), 61.12 (1C, C-20, –OCH_3_), 62.56 (1C, C-19, –OCH_3_), 108.10 (1C, C-17), 119.09 (1C, C-12), 122.42 (1C, C-16), 126.02 (2C, C-3, C-5), 127.44 (1C, C-4), 136.11 (2C, C-2, C-6), 142.13 (1C, C-15), 149.98 (1C, C-9), 153.14 (1C, C-1), 155.23 (1C, C-14), 157.74 (1C, C-13), 159.07 (1C, C-11), 162.68 (1C, C-10). HREIMS *m*/*z* 558.2336 [M]^+^ (calcd for C_28_H_38_O_4_N_4_S_2_ 558.2334).

*3-((3,5-Di-tert-Butyl-4-hydroxybenzylthio)methyl)-4-(3,4,5-trimethoxybenzylideneamino)-1H-1,2,4-triazole-5(4H)-thione* (**5**). Prepared from 3,4,5-trimethoxybenzaldehyde (0.63 mmol, 0.123 gm), and recrystallized from 5:1 methanol-water to give 0.27 gm of white solid (76%). Mp 158–160 °C. IR (KBr), cm^−1^: ν = 3631, 3606 (free OH, BHP), 3447 (NH), 2840–2957 (C–H of CH_3_), 1608 (C=N), 1240 (C=S). ^1^H-NMR δ, ppm (Figure S3): 1.40 (s, 18H, 2 × *t*-Bu), 3.72 (s, 2H, H-7), 3.73 (2H, H-8), 3.90 (6H, H-18, H-20, 2 × OCH_3_), 3.91 (3H, H-19, –OCH_3_), 5.16 (1H, OH, BHP), 7.09 (2H, H-13, H-17), 7.10 (s, 2H, H-3, H-5), 10.15 (1H, C-11), 11.33 (1H, NH). ^13^C-NMR δ, ppm (Figure S11): 24.38 (1C, C-8), 30.35 (6C, 2 × –C(CH_3_)_3_), 34.41 (2C, 2 × –C(CH_3_)_3_), 36.54 (1C, C-7), 56.35 (2C, C-18, C-20), 61.14 (1C, C-19), 106.00 (2C, C-13, C-17), 126.00 (2C, C-3, C-5), 127.33 (1C, C-4), 127.64 (1C, C-12), 136.13 (2C, C-2, C-6), 142.05 (1C, C-15), 150.11 (1C, C-9), 153.18 (1C, C-1), 153.65 (2C, C-14, C-16), 161.52 (1C, C-11), 162.60 (1C, C-10). HREIMS *m*/*z* 558.2335 [M]^+^ (calcd for C_28_H_38_O_4_N_4_S_2_ 558.2334).

*4-(3,5-Di-tert-butyl-4-hydroxybenzylideneamino)-3-((3,5-di-tert-butyl-4-hydroxybenzylthio)methyl)-1H-1,2,4-triazole-5(4H)-thione* (**6**). Prepared from 3,5-di-tert-butyl-4-hydroxybenzaldehyde (0.63 mmol, 0.147 gm), recrystallized from 5:2 methanol-water to give 0.26 gm of white solid (69%). Mp 172–174 °C. IR (KBr), cm^−1^: ν = 3627–3605 (2OH, 2BHP), 2872–2954 (C–H of *t*-Bu), 1596–1581 (C=N), 1233 (C=S). ^1^H-NMR δ, ppm (Figure S4): 1.41 (s, 18H, 2 × *t*-Bu, BHP-A), 1.45 (s, 18H, 2 × *t*-Bu, BHP-B), 3.70 (s, 2H, H-8), 3.72 (s, 2H, H-7), 5.15 (1H, OH, BHP-A), 5.70 (1H, OH, BHP-B), 7.09 (s, 2H, H-3, H-5), 7.69 (s, 2H, H-13, H-17), 9.87 (1H, C-11), 10.60–10.79 (1H, NH). ^13^C-NMR δ ppm (Figure S12): 24.39 (1C, C-8), 30.18 (6C, 2 × –C(CH_3_)_3_, BHP-B), 30.36 (6C, 2 × –C(CH_3_)_3_, BHP-A), 34.40 (2C, 2 × –C(CH_3_)_3_, BHP-A), 34.47 (2C, 2 × –C(CH_3_)_3_, BHP-B), 36.55 (1C, C-7), 123.54 (1C, C-12), 126.00 (2C, C-3, C-5), 126.66 (2C, C-13, C-17), 127.43 (1C, C-4), 136.07 (2C, C-2, C-6), 136.65 (2C, C-14, C-16), 150.07 (1C, C-9), 153.14 (1C, C-1), 158.37 (1C, C-15), 162.65 (1C, C-10), 164.18 (1C, C-11). HREIMS *m*/*z* 596.3218 [M]^+^ (calcd for C_33_H_48_O_2_N_4_S_2_ 596.3219).

*4-(3,5-Di-tert-butyl-2-hydroxybenzylideneamino)-3-((3,5-di-tert-butyl-4-hydroxy benzylthio)methyl)-1H-1,2,4-triazole-5(4H)-thione* (**7**). Prepared from 3,5-di-*tert*-butyl-2-hydroxybenzaldehyde (0.63 mmol, 0.147 gm), recrystallized from 5:2 methanol-water to give 0.25 gm of white solid (66%). Mp 126–128 °C. IR (KBr), cm^−1^: ν = 3609–3633 (2OH, 2BHP), 2869–2954 (C–H of *t*-Bu), 1610, 1575 (C=N), 1231 (C=S). ^1^H-NMR δ, ppm (Figure S5): 1.31 (s, 9H, *t*-Bu-C16, B) 1.42 (s, 18H, 2 × *t*-Bu, BHP-A), 1.44 (s, 9H, *t*-Bu-C14, B), 3.65 (s, 2H, H-8), 3.70 (s, 2H, H-7), 5.17 (1H, OH, A), 7.12 (s, 2H, H-3, H-5), 7.19, 7.20 (d, 1H, H-15, ^4^*J* = 2 Hz), 7.52, 7.53 (d, 1H, H-17, ^4^*J* = 2 Hz), 9.96 (1H, C-11), 10.61 (1H, OH, B), 11.24 (1H, NH). ^13^C-NMR δ, ppm (Figure S13): 24.47 (1C, C-8), 29.50 (3C, –C(CH_3_)_3_, o-OH, B), 30.37 (6C, 2 × –C(CH_3_)_3_, A), 31.46 (3C, –C(CH_3_)_3_, p-OH, B), 34.33 (1C, –C(CH_3_)_3_, p-OH, B), 34.43 (2C, 2 × –C(CH_3_)_3_, A), 35.30 (1C, –C(CH_3_)_3_, o-OH, B), 36.35 (1C, C-7), 115.35 (1C, C-12), 126.03 (2C, C-3, C-5), 127.11 (1C, C-4), 128.13 (1C, C-17), 130.20 (1C, C-15), 136.18 (2C, C-2, C-6), 137.56 (1C, C-14), 141.92 (1C, C-16), 148.58 (1C, C-9), 153.22 (1C, C-1), 157.35 (1C, C-13), 162.98 (1C, C-10), 168.69 (1C, C-11). HREIMS *m*/*z* 596.3228 [M]^+^ (calcd for C_33_H_48_O_2_N_4_S_2_ 596.3219).

*4-(4-(Benzyloxy)benzylideneamino)-3-((3,5-di-tert-butyl-4-hydroxybenzylthio)methyl)-1H-1,2,4-triazole-5(4H)-thione* (**8**). Prepared from 4-(benzyloxy)benzaldehyde (0.63 mmol, 0.133 gm), and recrystallized from ethanol to give 0.30 gm of white solid (83%). Mp. 192–194 °C. IR (KBr), cm^−1^: ν = 3635 (free OH, BHP), 2910–2948 (C–H of *t*-Bu, –CH_2_–O), 1598, 1577 (C=N), 1236 (C=S). ^1^H-NMR δ, ppm (Figure S6): 1.41 (s, 18H, 2 × *t*-Bu, BHP), 3.713 (s, 2H, H-7), 3.717 (s, 2H, H-8),5.13 (2H, H-18) 5.16 (1H, OH), 7.02, 7.04 (d, 2H, H-13, H-17, 3*J* = 8.6 Hz), 7.10 (2H, H-3, H-5), 7.40 (m, 1H, H-22), 7.37–7.39 (m, 2H, H-21, H-23), 7.42–7.44 (m, 2H, H-20, H-24), 7.78, 7.80 (d, 2H, H-14, H-16, ^3^*J* = 8.8 Hz), 10.13 (1H, C-11), 11.24 (1H, NH). ^13^C-NMR δ, ppm (Figure S14): 24.30 (1C, C-8), 30.36 (6C, 2 × –C(CH_3_)_3_, BHP), 34.42 (2C, 2 × –C(CH_3_)_3_), 36.53 (1C, C-9), 70.26 (1C, C-18), 115.40 (2C, C-13, C-17), 125.28 (1C, C-12), 126.00 (2C, C-3, C-5), 127.40 (1C, C-19), 127.56 (2C, C-20, C-24), 128.34 (1C, C-22), 128.80 (2C, C-21, C-23), 130.85 (2C, C-14, C-16), 136.11 (2C, C-2, C-6), 136.26 (1C, C-4), 150.17 (1C, C-9), 153.15 (1C, C-1), 161.39 (1C, C-11), 162.43 (1C, C-15), 162.65 (1C, C-10). HREIMS *m*/*z* 574.2408 [M]^+^ (calcd for C_32_H_38_O_2_N_4_S_2_ 574.2436).

*4-(Biphenyl-4-ylmethyleneamino)-3-((3,5-di-tert-butyl-4-hydroxybenzylthio)methyl)-1H-1,2,4-triazole-5(4H)-thione* (**9**). Prepared from 4-phenylbenzaldehyde (0.63 mmol, 0.11 gm), and recrystallized from 5:2 ethanol–water to give 0.29 gm of white solid (85%). Mp. 185–187 °C. IR (KBr), cm^−1^: ν = 3630 (free OH, BHP), 3421 (NH), 2913–2951 (C–H of *t*-Bu), 1602 (C=N), 1237 (C=S). ^1^H-NMR δ, ppm (Figure S7): 1.41 (s, 18H, 2 × *t*-Bu, BHP), 3.72 (s, 2H, H-8), 3.75 (s, 2H, H-7), 5.16 (1H, OH), 7.11 (2H, H-3, H-5), 7.39 (t, 1H, H-21, ^3^*J* = 8 Hz), 7.47 (t, 2H, H-20, H-22, ^3^*J* = 7.6, ^3^*J* = 7.6 Hz), 7.62 (d, 2H, H-19, H-23, ^3^*J* = 8 Hz), 7.69 (d, 2H, H-14, H-16, 3*J* = 8 Hz), 7.91 (d, 2H, H-13, H-17, 3*J* = 8 Hz). 10.43 (1H, H-11), 10.89 (1H, NH). ^13^C-NMR δ, ppm (Figure S15): 24.31 (1C, C-8), 30.37 (6C, 2 × –C(CH_3_)_3_, BHP), 34.42 (2C, 2 × –C(CH_3_)_3_), 36.59 (1C, C-7), 126.01 (2C, C-3, C-5), 127.28 (2C, C-13, C-17), 127.37 (1C, C-4), 127.71 (2C, C-14, C-16), 128.27 (1C, C-21), 129.06 (2C, C-20, C-22), 129.40 (2C, C-13, C-17), 131.44 (1C, C-12), 136.13 (2C, C-2, C-6), 140.04 (1C, C-18), 145.35 (1C, C-15), 150.34 (1C, C-9), 153.18 (1C, C-1), 160.57 (1C, C-11), 162.78 (1C, C-10). HREIMS *m*/*z* 544.2324 [M]^+^ (calcd for C_31_H_36_N_4_O_2_S_2_ 544.2331).

*4-(4-Chlorobenzylideneamino)-3-((3,5-di-tert-butyl-4-hydroxybenzylthio)methyl)-1H-1,2,4-triazole-5(4H)-thione* (**10**). Prepared from 4-chlorobenzaldehyde (0.63 mmol, 0.088 gm), and recrystallized from 5:1 methanol–water to give 0.24 gm of white solid, yield (76%). Mp. 167–169 °C. IR (KBr), cm^−1^: ν = 3638, 3616 (OH, BHP), 2912 2950 (C–H of *t*-Bu), 1593, 1581 (C=N), 1223 (C=S), 1087 (C-Cl, stretching). ^1^H-NMR δ, ppm (Figure S8): 1.41 (s, 18H, 2 × *t*-Bu), 3.70 (s, 2H, H-7), 3.72 (s, 2H, H-8), 5.17 (1H, OH), 7.10 (s, 2H, H-3, H-5), 7.22, 7.44 (d, 2H, H-13, H-17, ^3^*J* = 8 Hz), 7.76, 7.78 (d, 1H, H-14, H-16, ^3^*J* = 8 Hz), 10.47 (s, 1H, H-11), 11.07 (b, 1H, NH). ^13^C-NMR δ, ppm (Figure S16): 24.28 (1C, C-8), 30.36 (6C, 2 × –C(CH_3_)_3_, 34.42 (2C, 2 × –C(CH_3_)_3_), 36.53 (1C, C-7), 125.99 (2C, C-3, C-5), 127.30 (1C, C-4), 129.44 (2C, C-13, C-17), 130.00 (2C, C-14, C-16), 131.11 (1C, C-12), 136.15 (2C, C-2, C-6), 138.72 (1C, C-15), 150.26 (1C, C-9), 153.18 (1C, C-1), 159.09 (1C, C-11), 162.67 (1C, C-10). HREIMS *m*/*z* 502.1629 [M]^+^ (calcd for C_25_H_31_ON_4_ClS_2_ 502.1628).

### 3.4. Antioxidant Assay

#### In Vitro DPPH Free Radical Scavenging Assay

The DPPH radical scavenging assay was carried out according to the literature [69] with some modifications. Briefly, 1.0 mL of DPPH solution (200 µM in DMSO) was added to a range of various sample concentrations (100, 10, 1, 0.1 and 0.01 µM). Then, 22.03–47.76 mg (1 × 10^−4^ M) of the test compound was dissolved in 1.0 mL DMSO (100%) as a stock solution. This stock solution was then diluted to a range of final extraction concentrations of 100, 10, 1, 0.1 and 0.01 µM. As a negative control, the same DPPH concentration in DMSO without sample was used. Each assay was carried out in triplicate. The mixture was then incubated in the dark for 60 min at room temperature. Absorbance at 570 nm for each sample was then measured. BHT was used as a positive control. The free radical scavenging activity of the compounds was calculated as a percentage of radical inhibition using the following formula. Percentage of Inhibition (%) = ((Ac−As)/Ac) × 100, where As = Absorbance of the compounds/positive control and Ac = Absorbance of control (DPPH solution and DMSO). To determine the concentration required to achieve 50% inhibition (IC_50_) of the DPPH radical, the percentage of DPPH inhibition for each compound was plotted against the extract concentration.

## 4. Conclusions

SAR and rational design approaches involved integrating multiple radical-scavenging ability and manifold pharmacological activities in designing hybrid compounds with significantly enhanced radical-scavenging capacity and pharmacophores of the well-known antioxidant BHT. We have synthesized novel derivatives of the antioxidant BHT which are Schiff base-1,2,4-triazoles, as a new generation of MPAO. Experimental data of the present study revealed that the synthesized novel Schiff base-1,2,4-triazoles improved the antioxidant capacity of BHT (25.23%) measured as the inhibition of DPPH to three-fold higher in compounds **6** (89.52%) and **7** (79.61%). The PASS and MPAO design approaches can be effectively used to determine lead compounds lacking undesirable side effects. PASS prediction of the Schiff base-1,2,4-triazoles were Pa < 0.5, which is confirmed experimentally, thus, there is a more than 50% chance that the Schiff base-1,2,4-triazole structures have not been reported with this activity, making them possible valuable lead compounds. These interesting results have stimulated us to consider BHT as a standard platform for new synthetic antioxidant projects. The products could play a vital role in repairing cellular damage, preventing various human diseases and in medical therapeutic application.

## Figures and Tables

1. Phenolic group
2. Two *tert*-butyl substituents flanking the OH group
3. Primary antioxidant, acting as chain-breaking antioxidant
4. –CH_2_– that stabilizes and decreases the BDE value of OH
5. Secondary antioxidant, hydroperoxide decomposers
6. N–H exchangeable proton
7. Triazole, inhibitor ring
8. Substituted phenyl to increase the free radical stabilization
9. Electron donating group
10. Thiourea (free radical scavenger system)
11. Imino group to extend free radical delocalization

**Table 1:** PASS prediction of the synthesized compounds **1**, **3**–**10** and BHT.

Mode of Biological Activity	1		3		4		5		6		7		8		9		10		BHT	
	Pa	Pi	Pa	Pi	Pa	Pi	Pa	Pi	Pa	Pi	Pa	Pi	Pa	Pi	Pa	Pi	Pa	Pi	Pa	Pi
Lipid peroxidase inhibitor	0.44	0.02	0.71	0.00	0.52	0.01	0.46	0.02	0.38	0.03	0.40	0.03	0.36	0.04	0.34	0.05	0.24	0.09	0.72	0.005
Antioxidant	0.41	0.01	0.41	0.01	0.29	0.02	0.31	0.02	0.30	0.02	0.29	0.02	0.26	0.03	0.27	0.02	0.25	0.03	0.58	0.005
Free radical scavenger	0.51	0.00	-	-	0.14	0.13	0.17	0.09	-	-	-	-	-	-	-	-		-	0.51	0.010
Lipoxygenase inhibitor	0.37	0.00	0.21	0.01	0.17	0.02	0.18	0.02	0.18	0.02	0.17	0.02	0.22	0.01	0.18	0.02	0.16	0.02	0.56	0.004
5-Lipoxygenase inhibitor	0.28	0.00	0.13	0.01	0.11	0.02	0.11	0.02	0.18	0.02	0.10	0.02	0.14	0.01	0.11	0.02	0.10	0.02	0.47	0.004

Pa—probability ‘to be active’; Pi—probability ‘to be inactive’.

**Table 2:** Preparation of 4-amino-3-((3,5-di-*tert*-butyl-4-hydroxybenzylthio)methyl)-1*H*-1,2,4-triazole-5(4*H*)-thione (**3**).

Entry	Compound 1 Mole	Thiocarbohydrazide Mole	Time h	Temperature °C	Yield (%)
1	1	1	3	90–120	24
2	1	1.25	3	125–130	70
3	1	1.25	3	155–160	45
4	1	1.5	3	125–130	90
5	1	2	3	125–130	90
6	1	1.5	3	145–150	65

**Table 3:** IC_50_ values and maximum inhibition of activity at 100 µM of the DPPH radical scavenging and lipid peroxidation inhibition assays.

Compound	DPPH IC_50_^a^ Values (µM) ± S.E.M ^b^	Radical Scavenging Max. Inhibition% ± S.E.M
**4**	64.04 ± 0.32	68.13 ± 0.27
**5**	58.26 ± 0.63	74.32 ± 0.14
**6**	46.13 ± 0.31	89.52 ± 0.09
**7**	52.80 ± 0.58	79.61 ± 0.23
**8**	67.59 ± 0.27	66.72 ± 0.15
**9**	68.04 ± 0.25	63.37 ± 0.13
**10**	72.16 ± 0.46	57.18 ± 0.21
BHT	>100 ^c^	25.23 ± 0.17

^a^ IC_50_: 50% effective concentration; ^b^ S.E.M: standard error of the mean; ^c^ did not reach 50% inhibition.

## Supplementary Materials

Supplementary materials can be accessed at: http://www.mdpi.com/1420-3049/21/7/874/s1.
