# Supplementary material for: Rational Design and Synthesis of New, High Efficiency, Multipotent Schiff Base-1,2,4-triazole Antioxidants Bearing Butylated Hydroxytoluene Moieties
Source: Molecules. 2016 Jun 28;21(7):847. doi: 10.3390/molecules21070847 (PMC6273539; doi:10.3390/molecules21070847)
Supplement: Supplementary file 1 [file molecules-21-00847-s001.pdf]

Supplementary Materials: Rational Design and Synthesis of New, High Efficiency, Multipotent Schiff Base-1,2,4-triazole Antioxidants Bearing Butylated Hydroxytoluene Moieties

Wageeh A Yehye, Noorsaadah Abdul Rahman , Omar Saad, Azhar Ariffin, Sharifah Bee Abd Hamid, Abeer A. Alhadi , Farkaad A. Kadir, Marzieh Yaeghoobi and Abdulsalam A. Matlob

Appendix A

^{1}H NMR

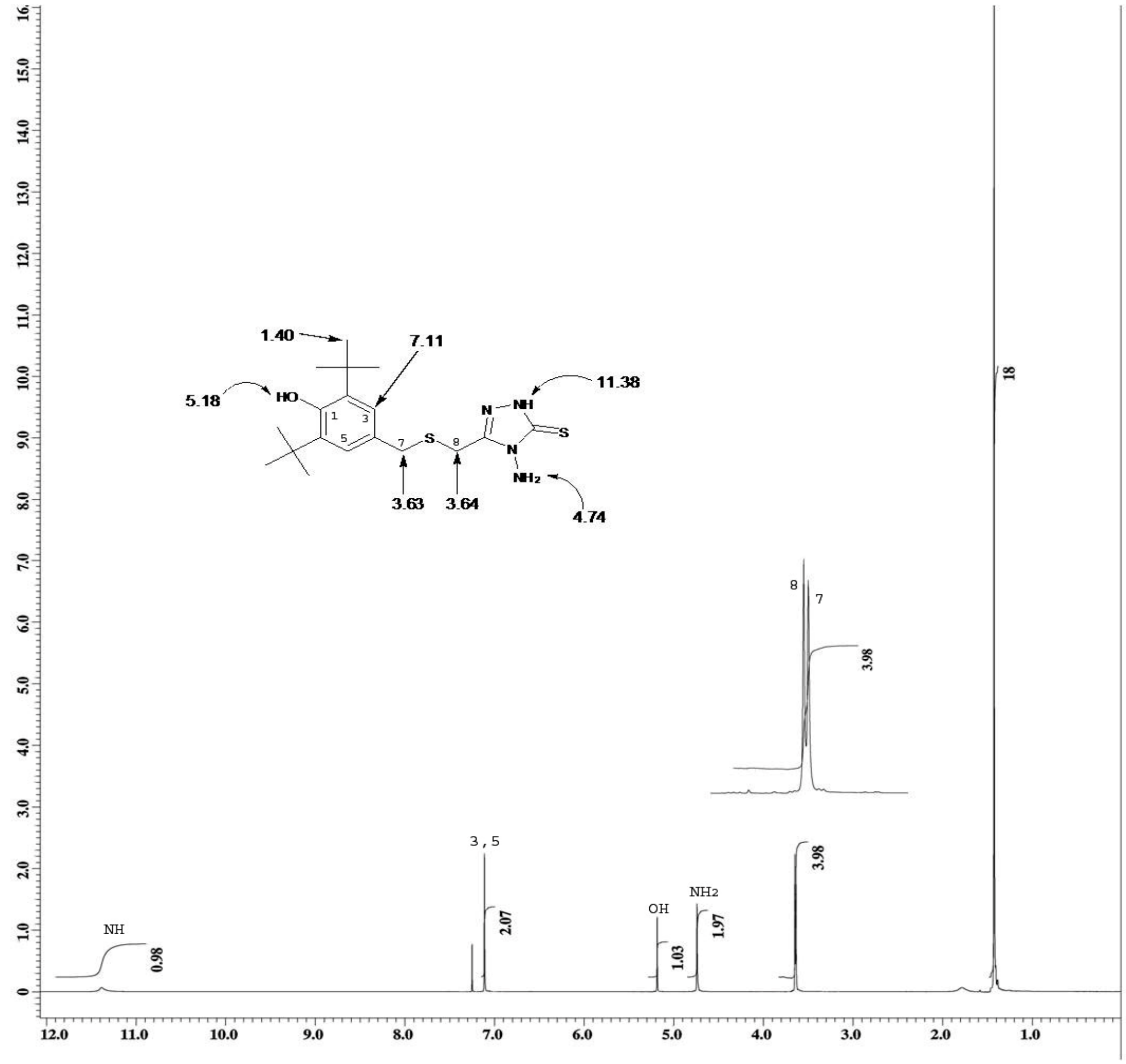

Figure S1. ^{1}H spectrum ($CDCl_3$, 400 MHz) of **3**.

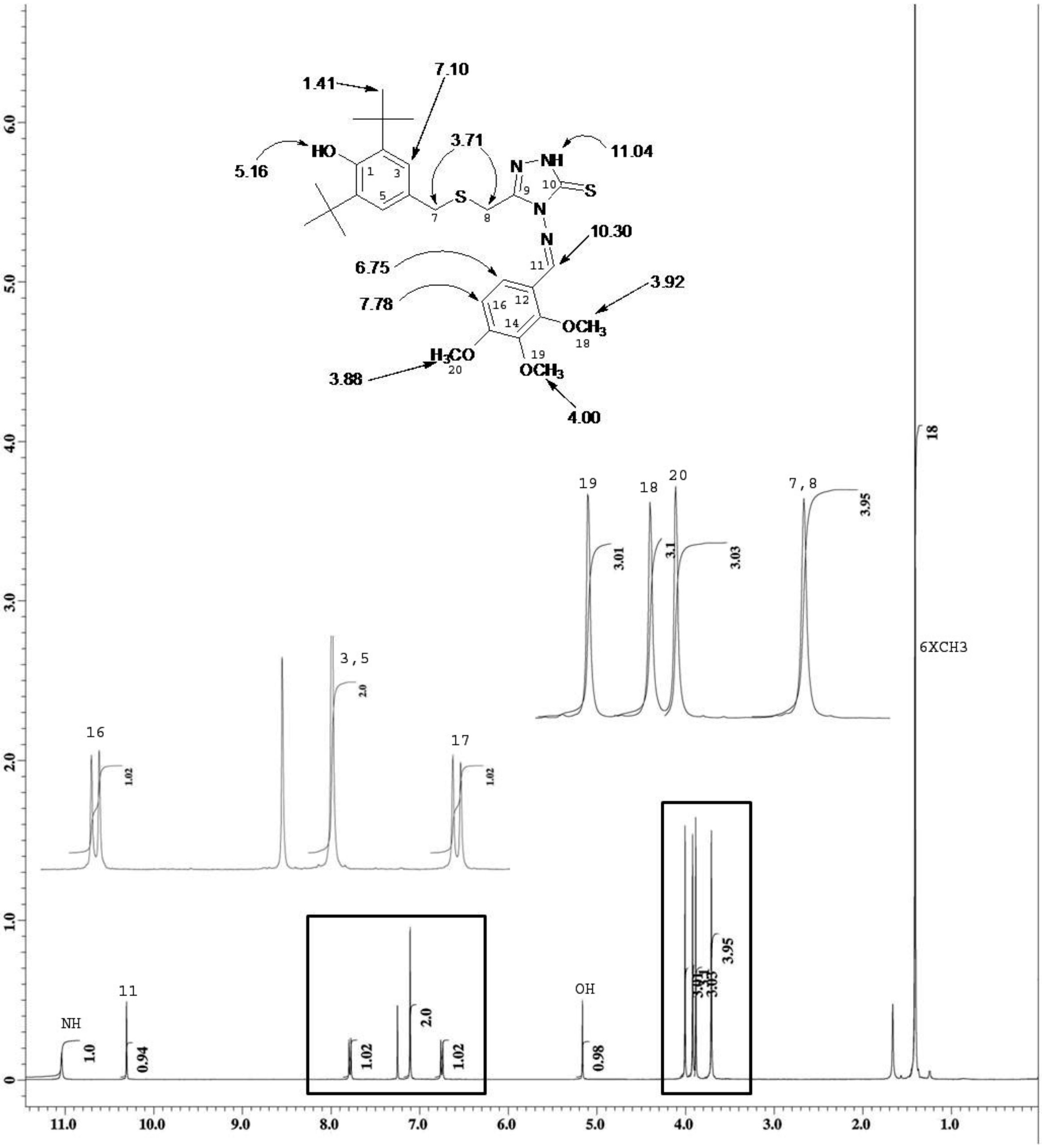

Figure S2. ^{1}H spectrum ($CDCl_3$, 400 MHz) of **4**.

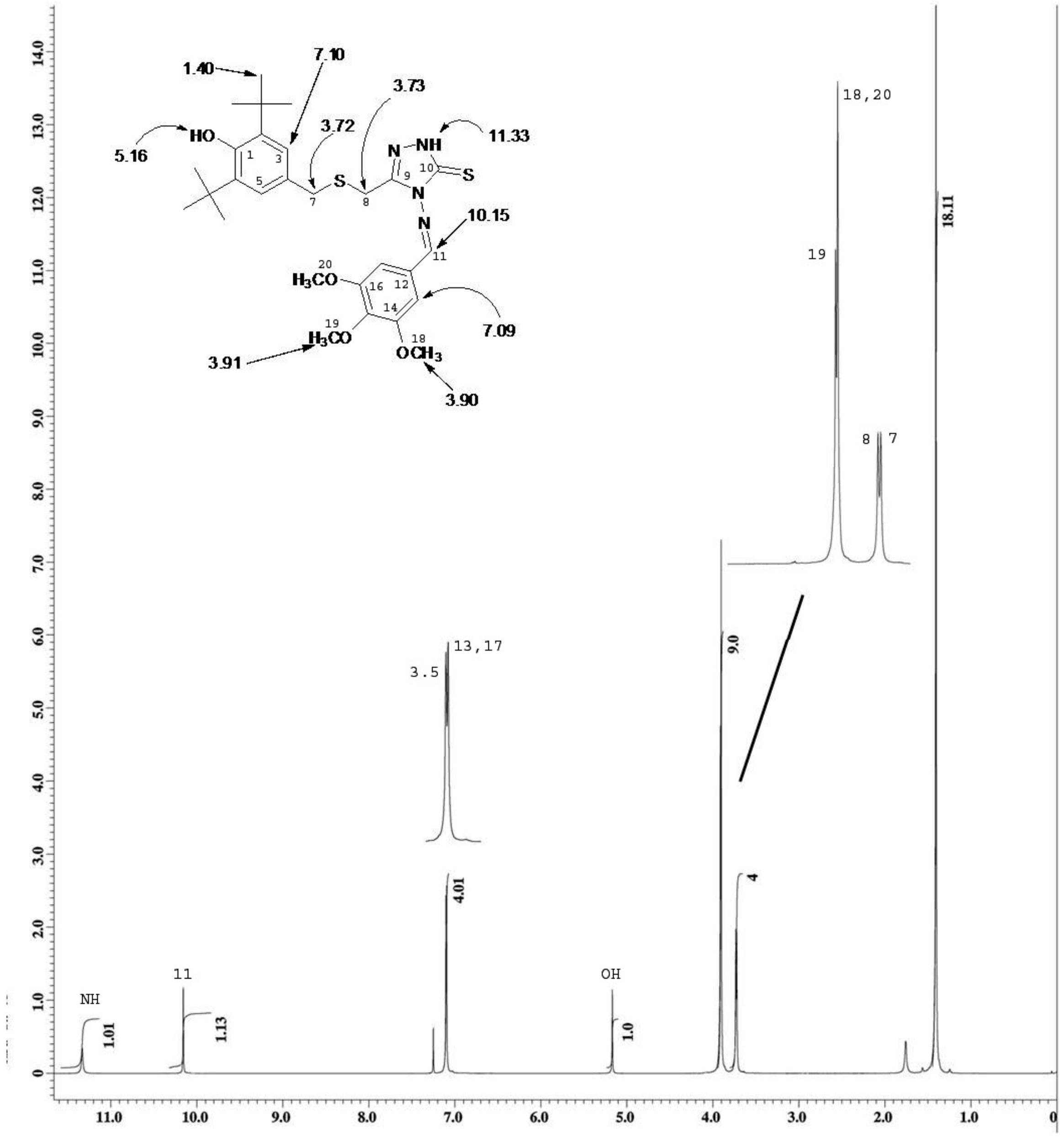

Figure S3. ^{1}H spectrum (CDCl3, 400 MHz) of **5**.

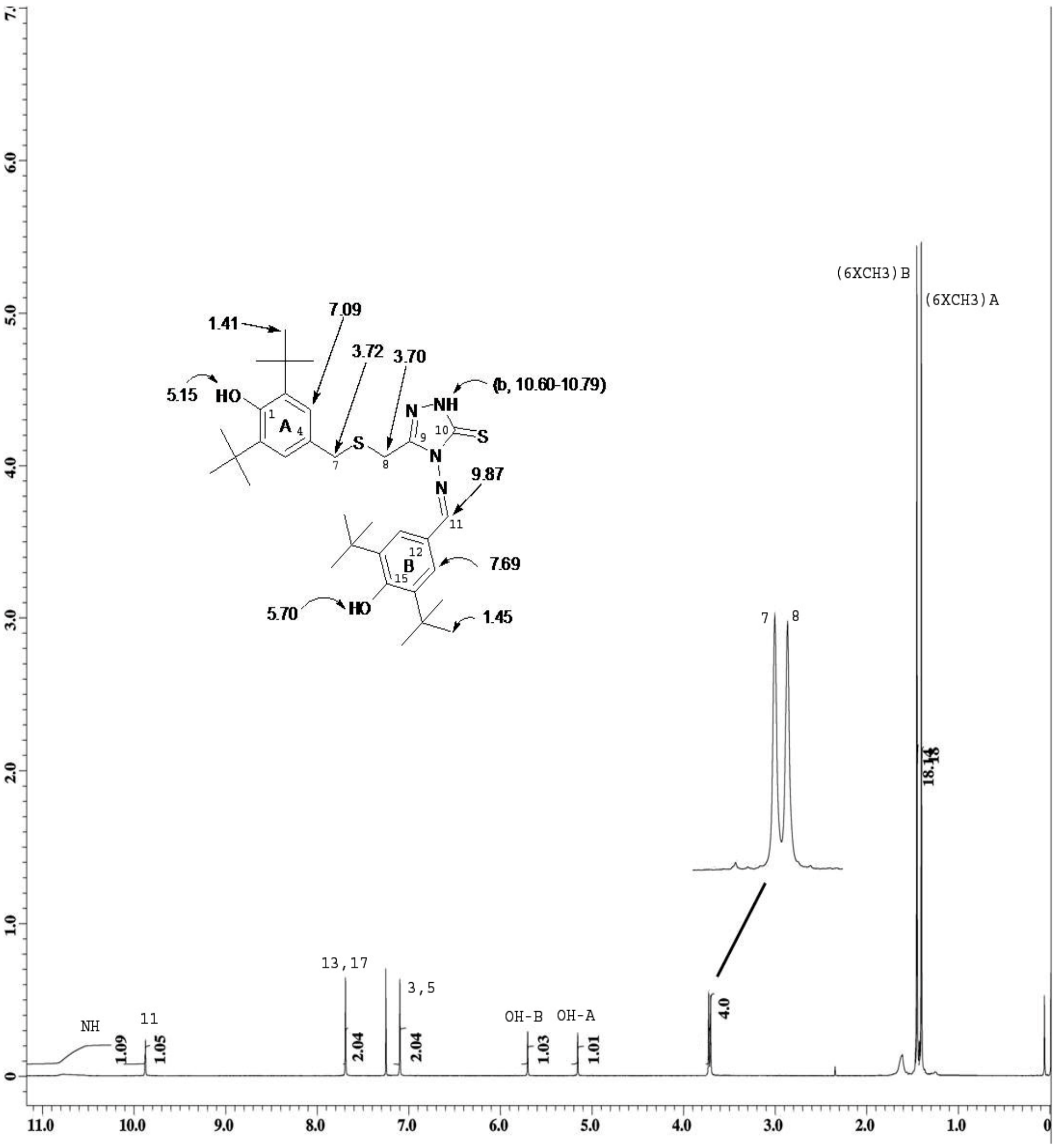

Figure S4. ^{1}H spectrum ($CDCl_3$, 400 MHz) of **6**.

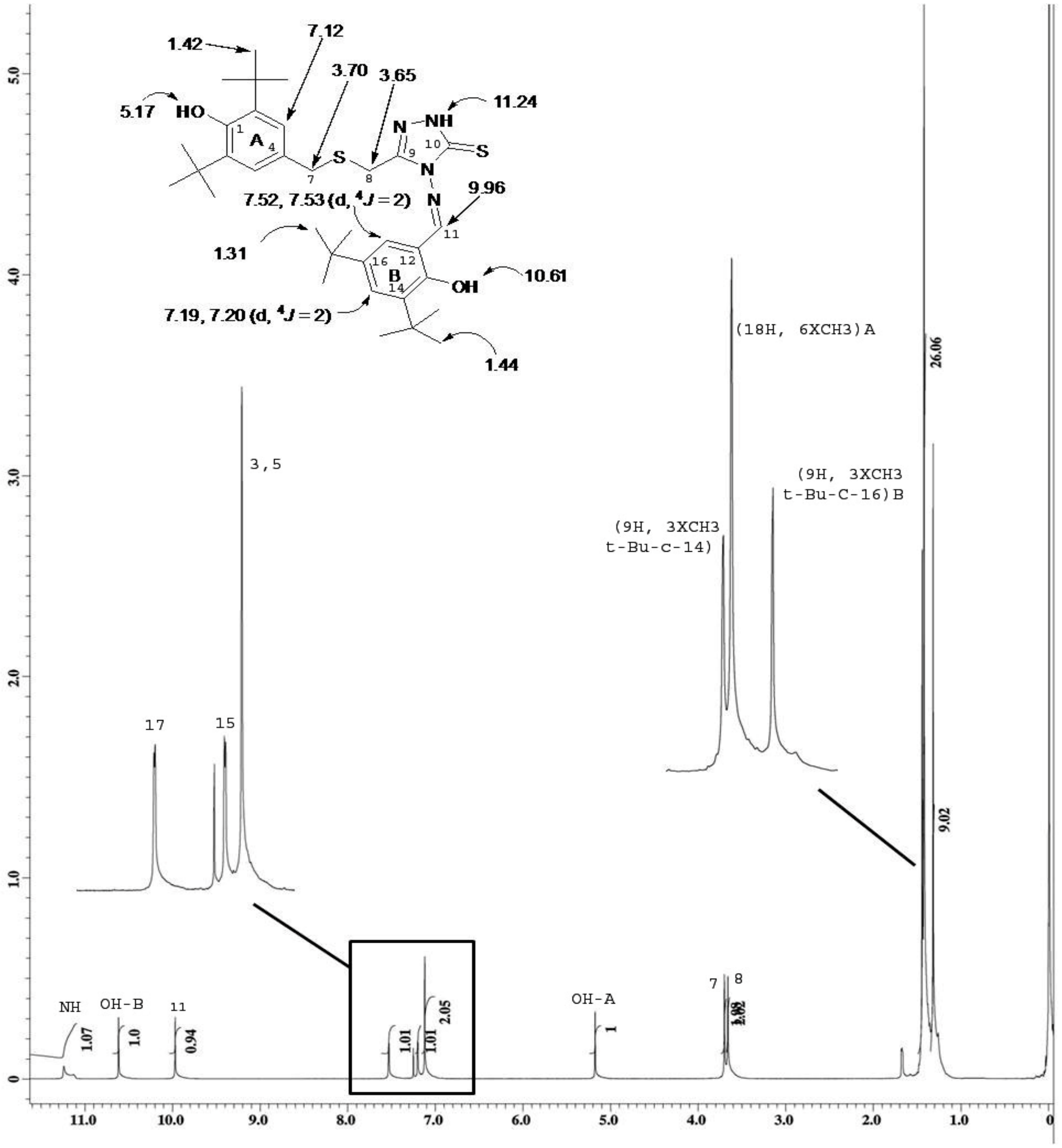

Figure S5. ^{1}H spectrum ($CDCl_3$, 400 MHz) of **7**.

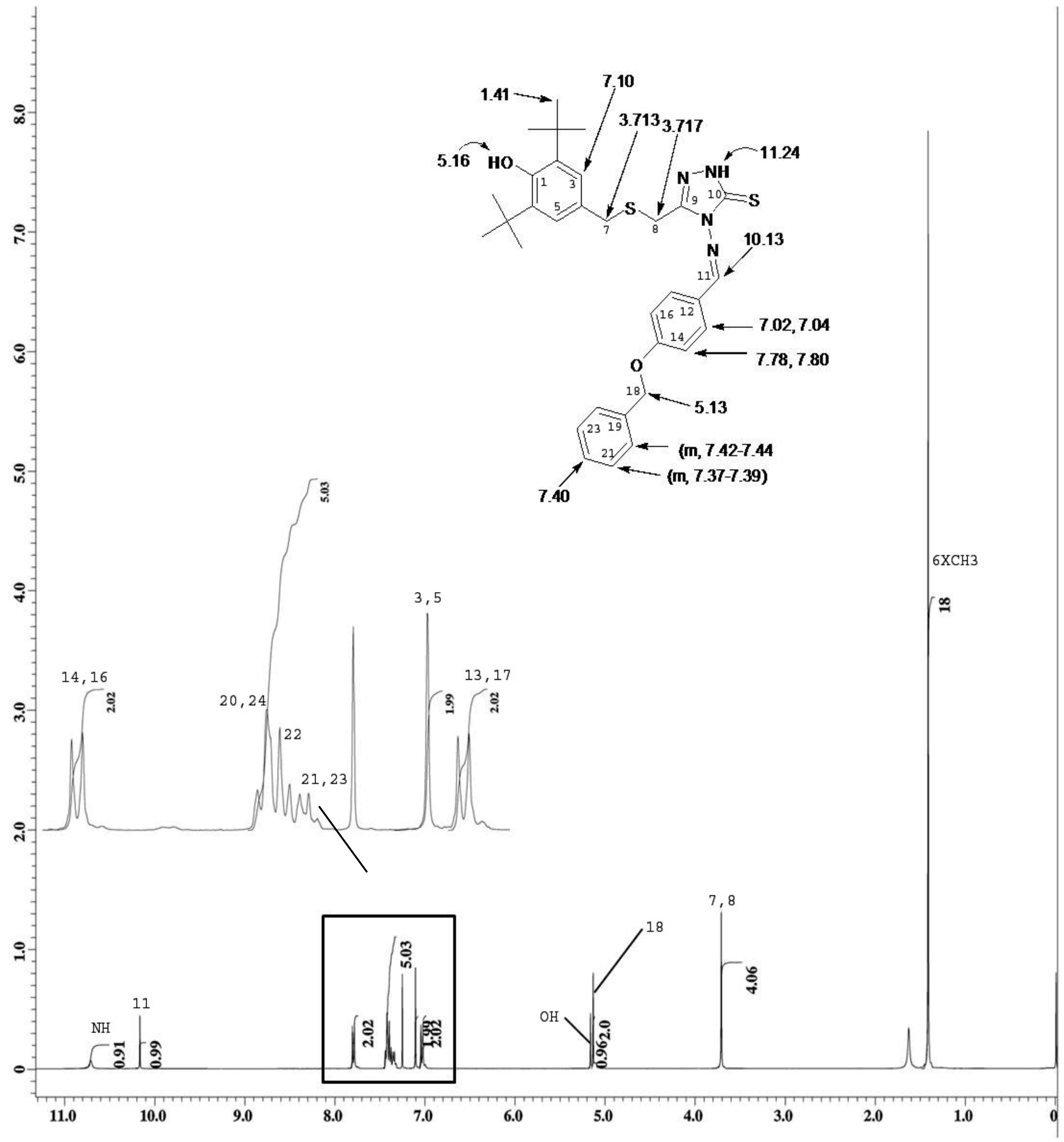

Figure S6. ^{1}H spectrum ($CDCl_3$, 400 MHz) of **8**.

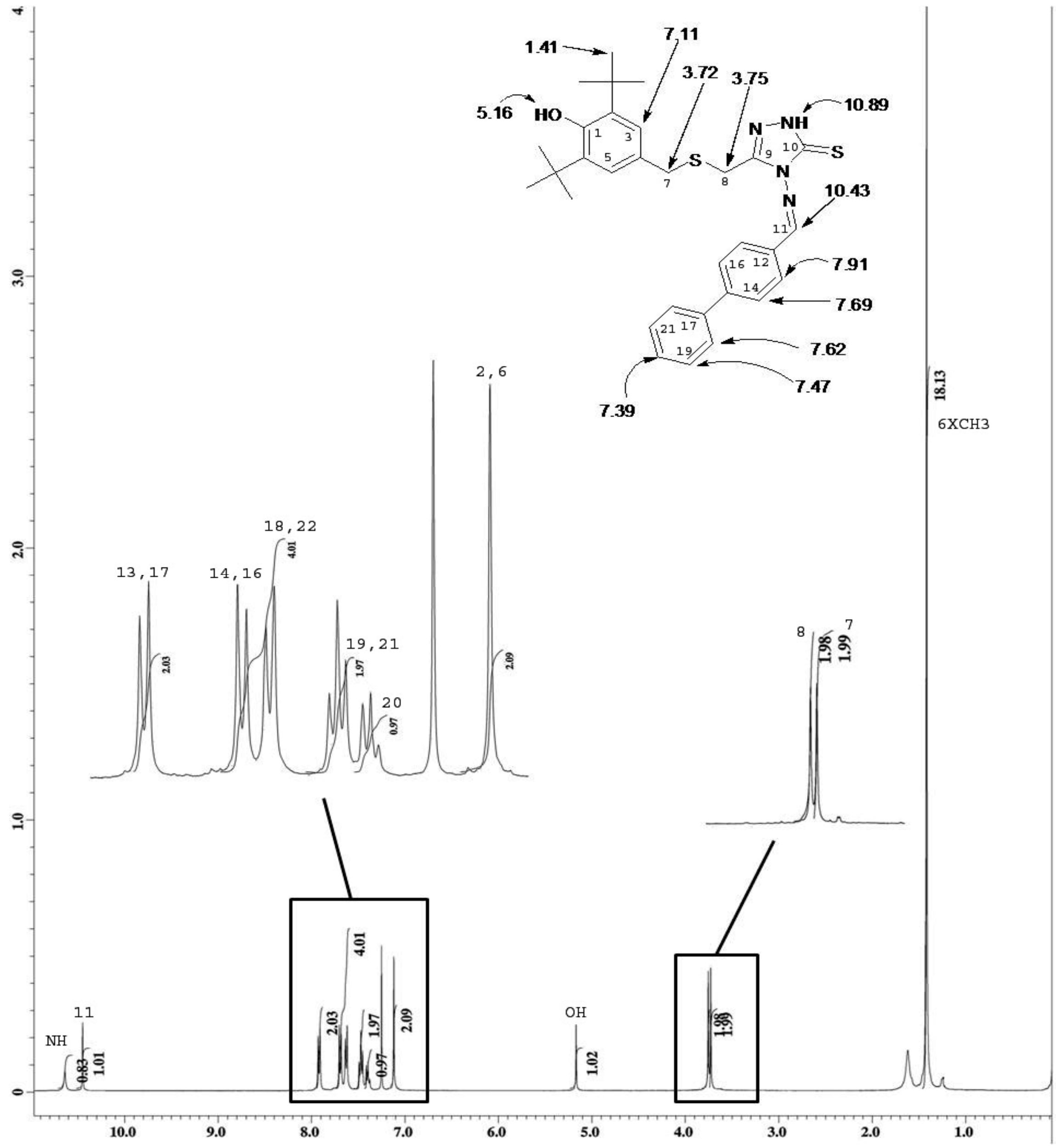

Figure S7. ^1H spectrum ($CDCl_3$, 400 MHz) of **9**.

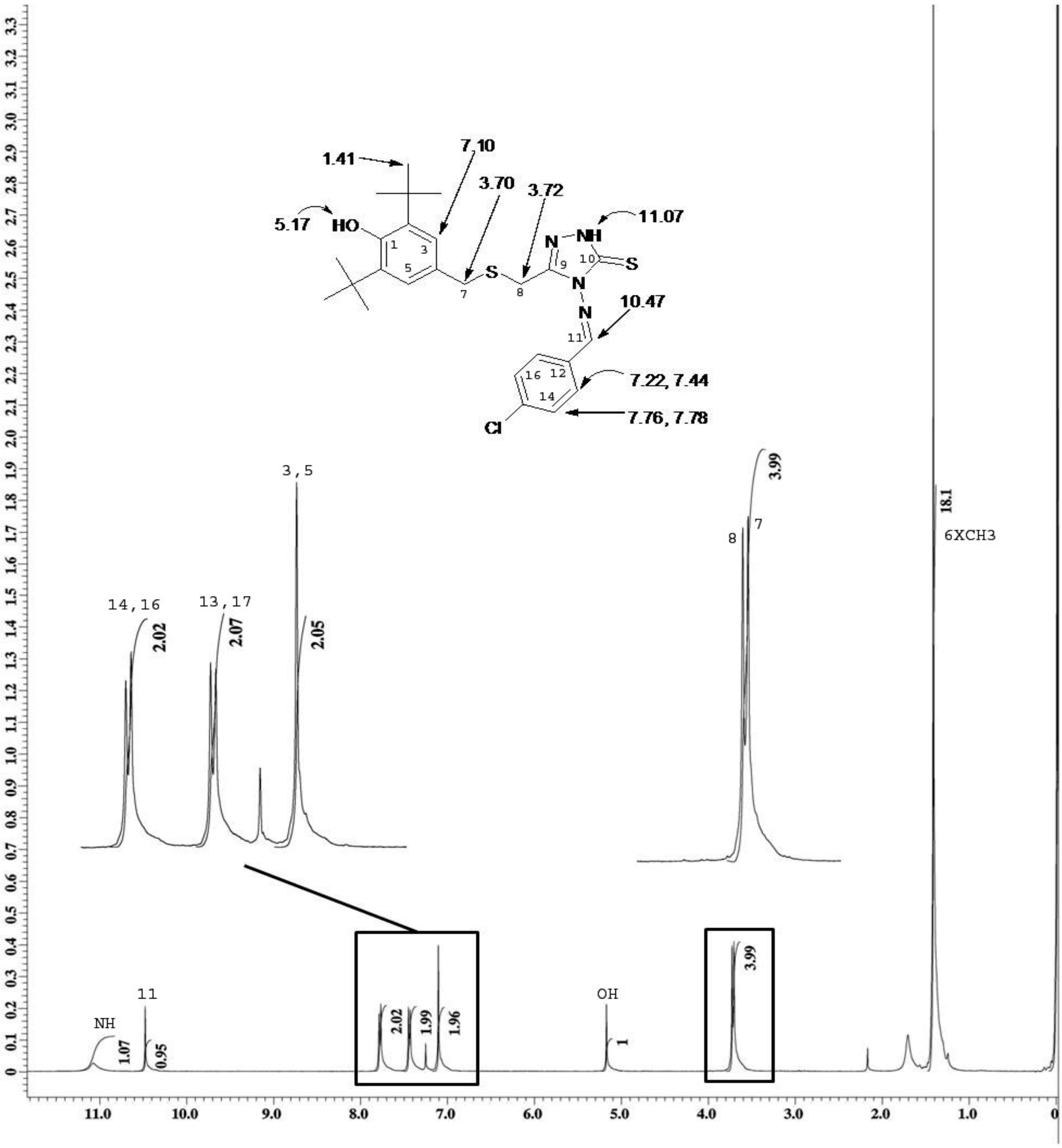

Figure S8. ^{1}H spectrum ($CDCl_3$, 400 MHz) of **10**.

Appendix B

^{13}C NMR

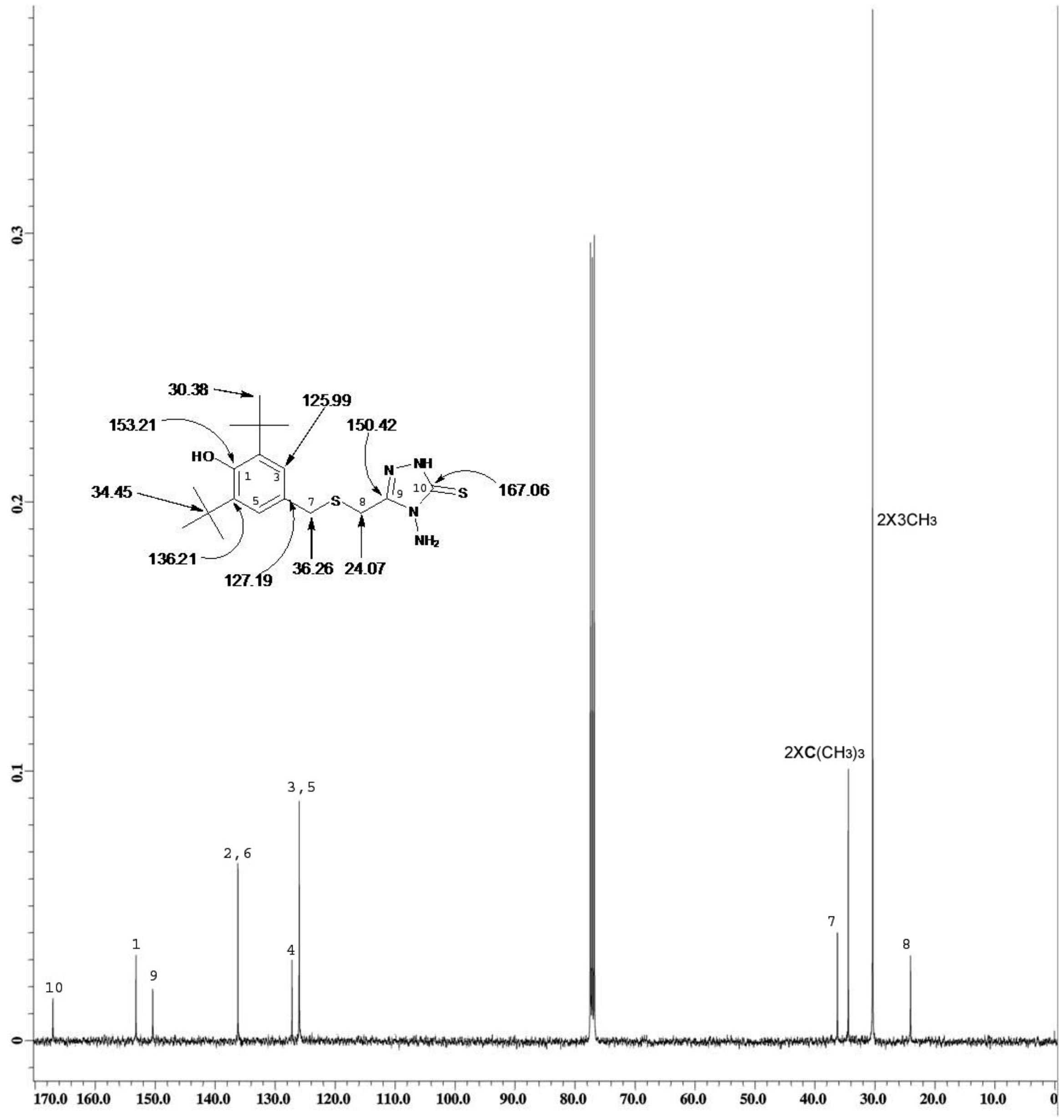

Figure S9. ^{13}C spectrum ($CDCl_3$, 100 MHz) of **3**.

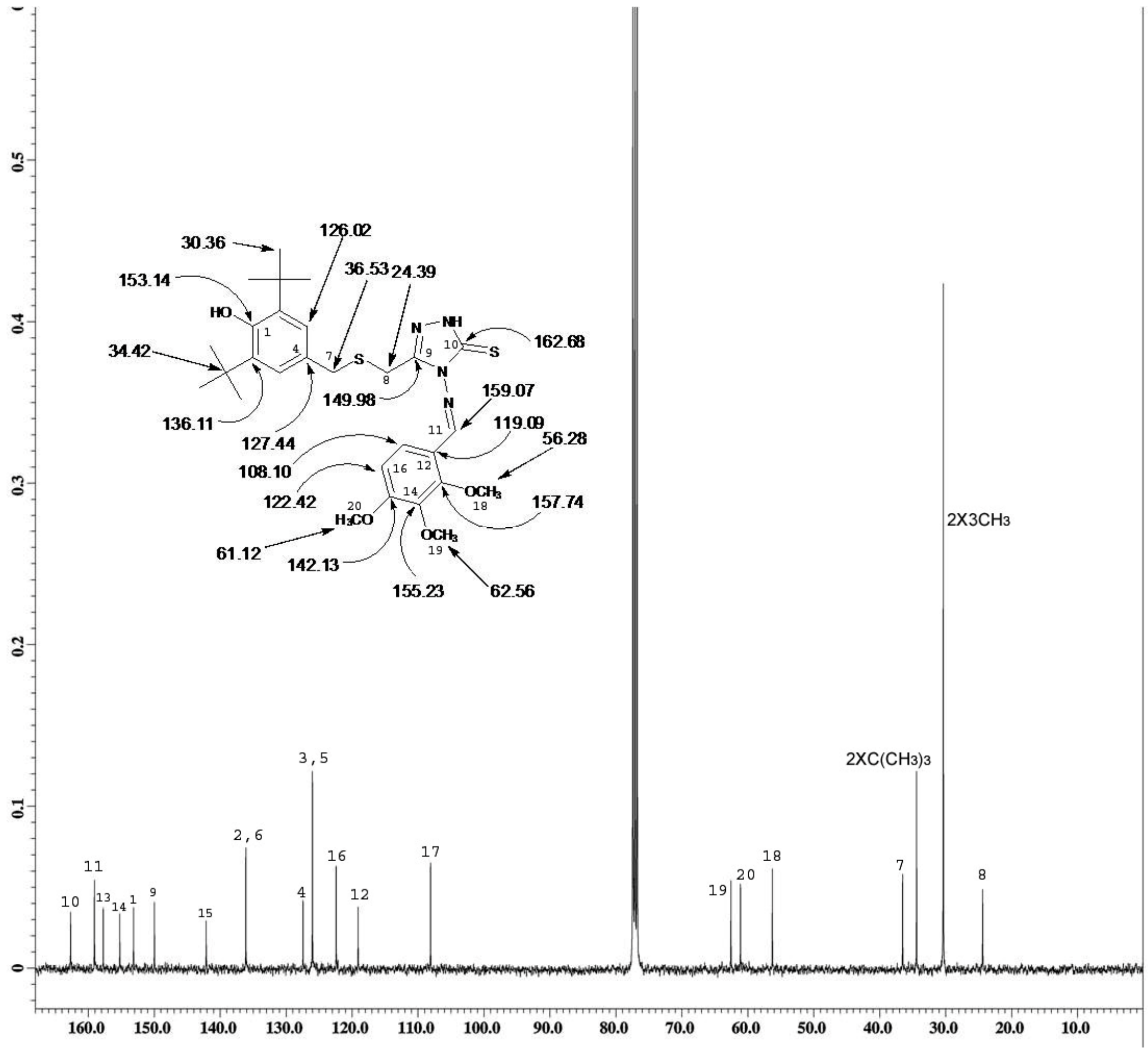

Figure S10. ^{13}C spectrum ($CDCl_3$, 100 MHz) of **4**.

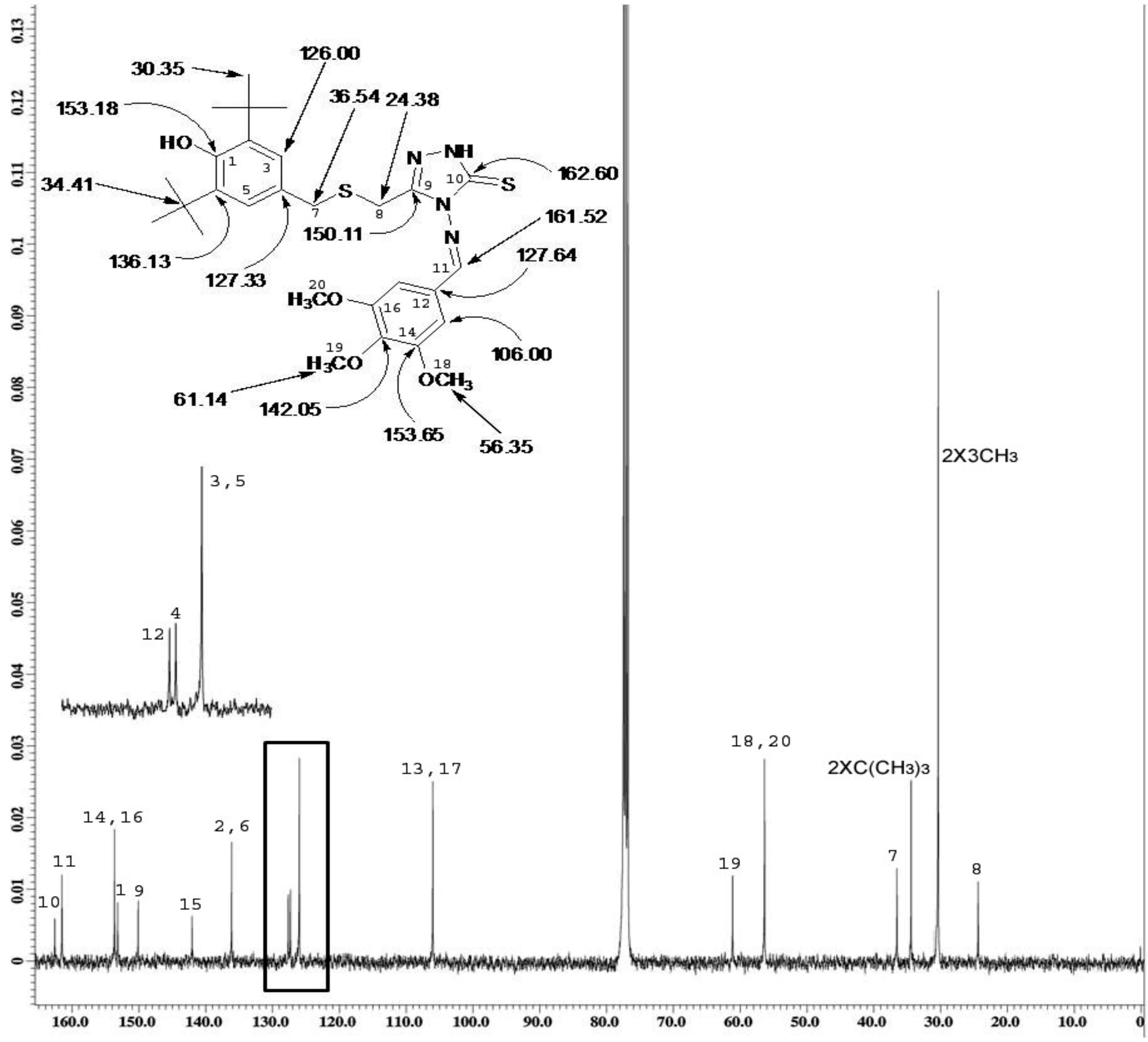

Figure S11. ^{13}C spectrum ($CDCl_3$, 100 MHz) of **5**.

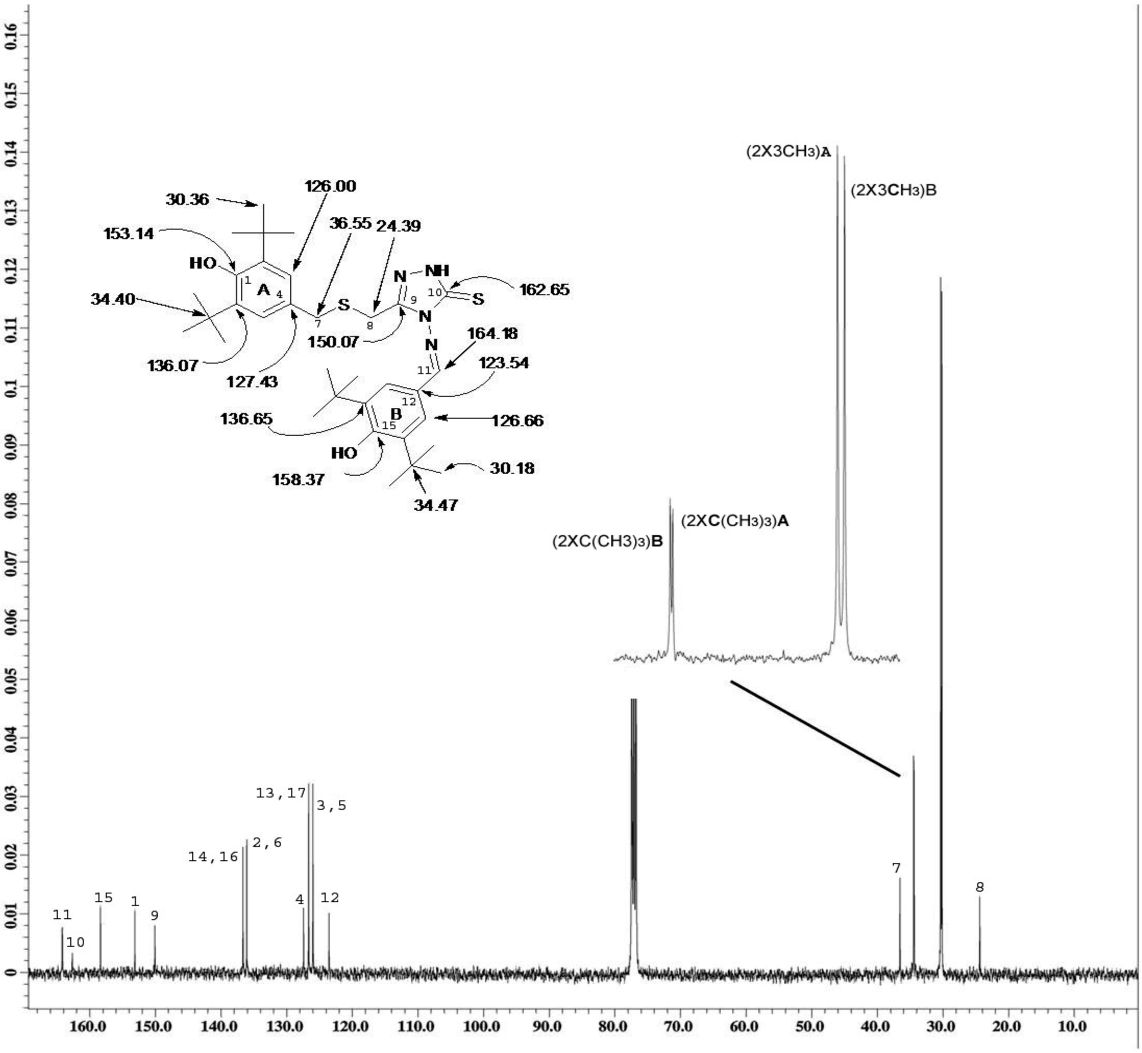

Figure S12. ^{13}C spectrum ($CDCl_3$, 100 MHz) of **6**.

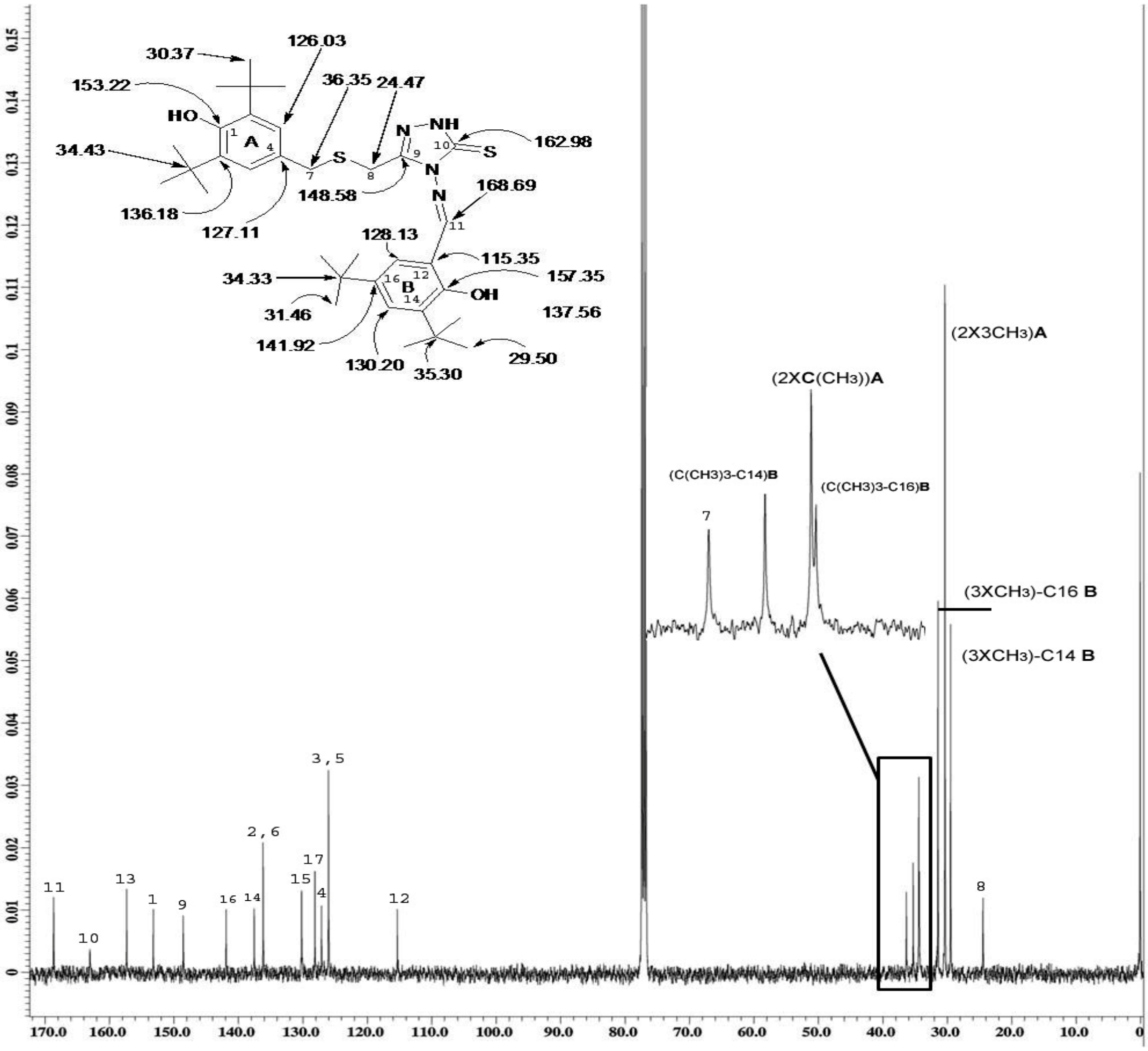

Figure S13. ^{13}C spectrum ($CDCl_3$, 100 MHz) of **7**.

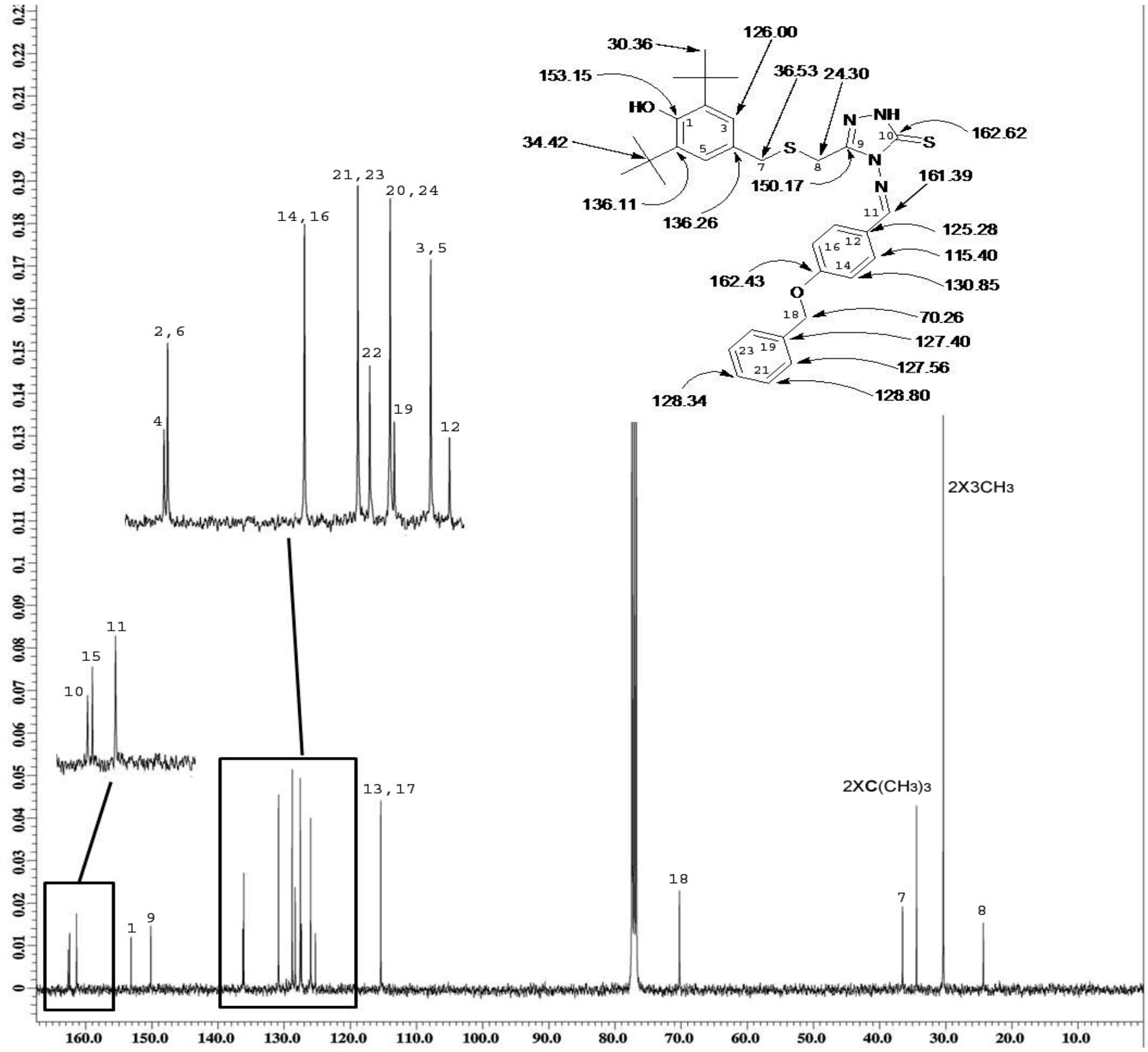

Figure S14. ^{13}C spectrum ($CDCl_3$, 100 MHz) of **8**.

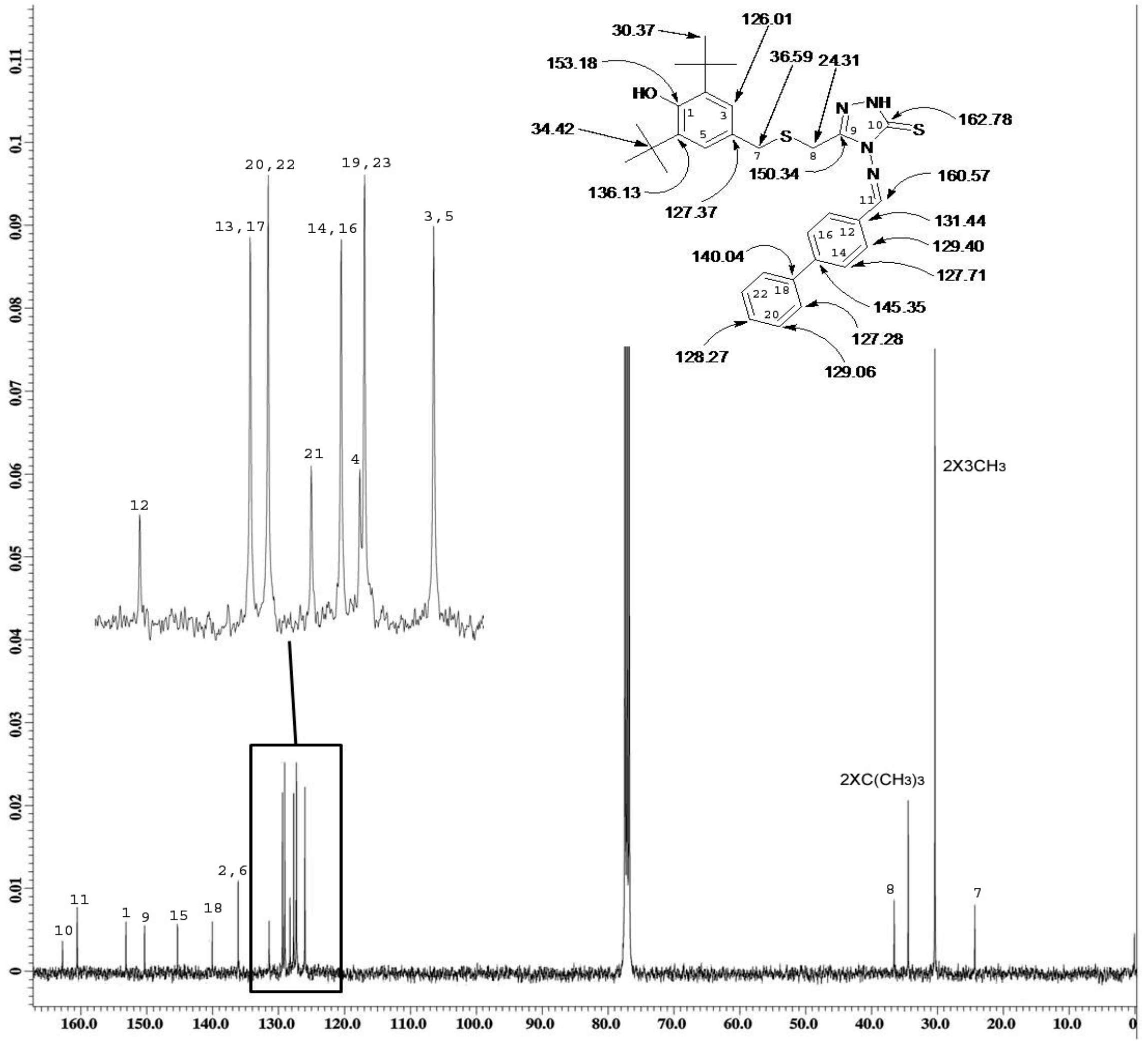

Figure S15. ^{13}C spectrum ($CDCl_3$, 100 MHz) of **9**.

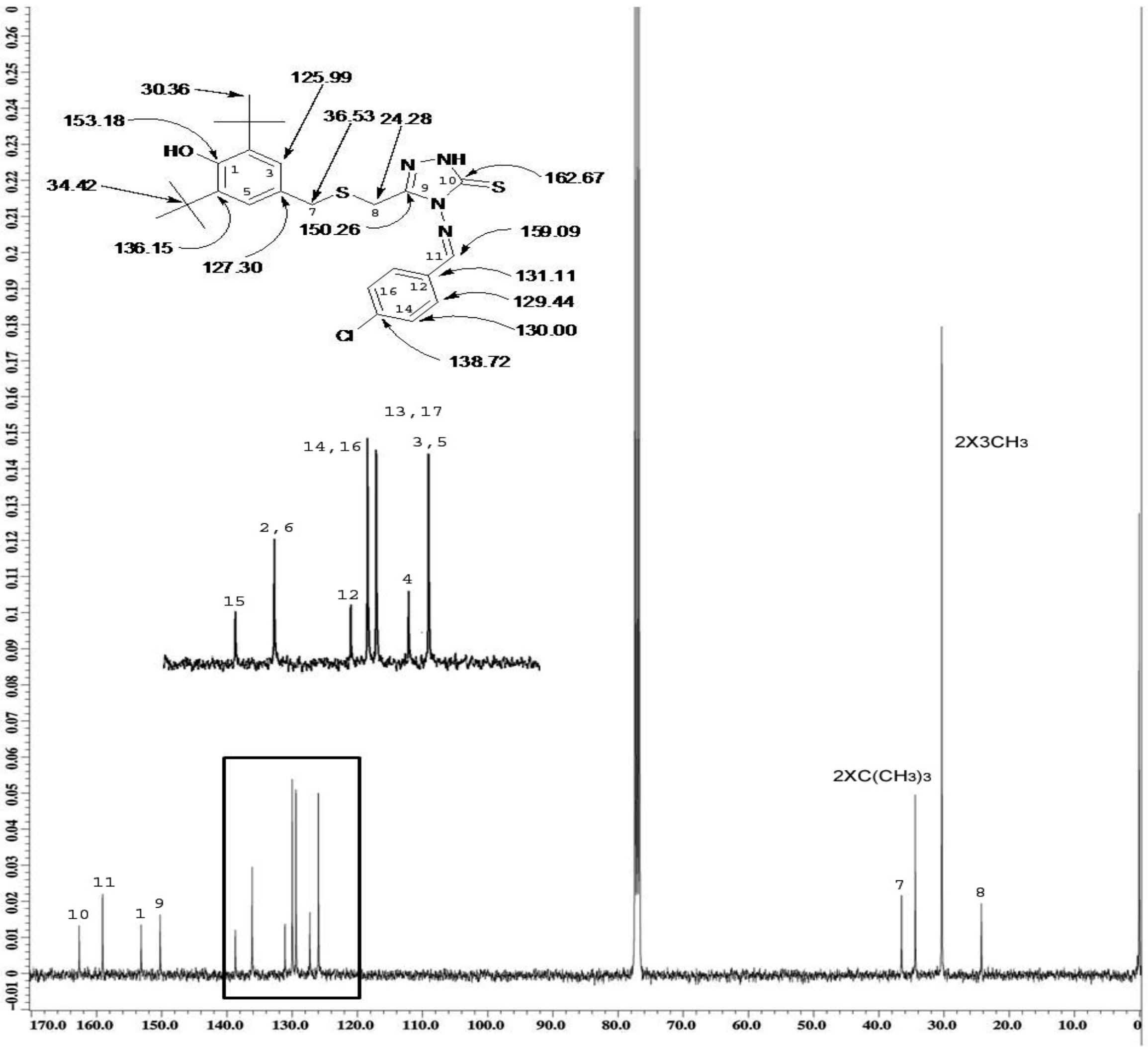

Figure S16. ^{13}C spectrum ($CDCl_3$, 100 MHz) of **10**.
